# Associations of fat mass and fat-free mass accretion in infancy with body composition and cardiometabolic risk markers at 5 years: The Ethiopian iABC birth cohort study

**DOI:** 10.1371/journal.pmed.1002888

**Published:** 2019-08-20

**Authors:** Rasmus Wibaek, Dorte Vistisen, Tsinuel Girma, Bitiya Admassu, Mubarek Abera, Alemseged Abdissa, Marit E. Jørgensen, Pernille Kæstel, Kim F. Michaelsen, Henrik Friis, Jonathan C. K. Wells, Gregers S. Andersen

**Affiliations:** 1 Department of Nutrition, Exercise and Sports, University of Copenhagen, Copenhagen, Denmark; 2 Clinical Epidemiology, Steno Diabetes Center Copenhagen, Gentofte, Denmark; 3 Department of Paediatrics and Child Health, Jimma University, Jimma, Ethiopia; 4 Jimma University Clinical and Nutrition Research Partnership (JUCAN), Jimma University, Jimma, Ethiopia; 5 Department of Population and Family Health, Jimma University, Jimma, Ethiopia; 6 Department of Psychiatry, Jimma University, Jimma, Ethiopia; 7 Department of Laboratory Sciences and Pathology, Jimma University, Jimma, Ethiopia; 8 National Institute of Public Health, Southern Denmark University, Copenhagen, Denmark; 9 Childhood Nutrition Research Centre, UCL Great Ormond Street Institute of Child Health, London, United Kingdom; Cornell University, UNITED STATES

## Abstract

**Background:**

Accelerated growth in early childhood is an established risk factor for later obesity and cardiometabolic disease, but the relative importance of fat mass (FM) and fat-free mass (FFM) accretion is not well understood. We aimed to study how FM and FFM at birth and their accretion during infancy were associated with body composition and cardiometabolic risk markers at 5 years.

**Methods and findings:**

Healthy children born at term were enrolled in the Infant Anthropometry and Body Composition (iABC) birth cohort between December 2008 and October 2012 at Jimma University Specialized Hospital in the city of Jimma, Ethiopia. FM and FFM were assessed using air displacement plethysmography a median of 6 times between birth and 6 months of age. In 507 children, we estimated individual FM and FFM at birth and their accretion over 0–3 and 3–6 months of age using linear-spline mixed-effects modelling. We analysed associations of FM and FFM at birth and their accretion in infancy with height, waist circumference, FM, FFM, and cardiometabolic risk markers at 5 years using multiple linear regression analysis. A total of 340 children were studied at the 5-year follow-up (mean age: 60.0 months; girls: 50.3%; mean wealth index: 45.5 out of 100; breastfeeding status at 4.5 to 6 months post-partum: 12.5% exclusive, 21.4% almost exclusive, 60.6% predominant, 5.5% partial/none). Higher FM accretion in infancy was associated with higher FM and waist circumference at 5 years. For instance, 100-g/month higher FM accretion in the periods 0–3 and 3–6 months was associated with 339 g (95% CI: 243–435 g, *p <* 0.001) and 367 g (95% CI: 250–484 g, *p <* 0.001) greater FM at 5 years, respectively. Higher FM at birth and FM accretion from 0 to 3 months were associated with higher FFM and cholesterol concentrations at 5 years. Associations for cholesterol were strongest for low-density lipoprotein (LDL)–cholesterol, and remained significant after adjusting for current FM. A 100-g higher FM at birth and 100-g/month higher FM accretion from 0 to 3 months were associated with 0.16 mmol/l (95% CI: 0.05–0.26 mmol/l, *p* = 0.005) and 0.06 mmol/l (95% CI: 0.01–0.12 mmol/l, *p* = 0.016) higher LDL-cholesterol at 5 years, respectively. Higher FFM at birth and FFM accretion in infancy were associated with higher FM, FFM, waist circumference, and height at 5 years. For instance, 100-g/month higher FFM accretion in the periods 0–3 and 3–6 months was associated with 1,002 g (95% CI: 815–1,189 g, *p <* 0.001) and 624 g (95% CI: 419–829 g, *p <* 0.001) greater FFM at 5 years, respectively. We found no associations of FM and FFM growth with any of the other studied cardiometabolic markers including glucose, HbA1c, insulin, C-peptide, HOMA-IR, triglycerides, and blood pressure. Non-attendance at the 5-year follow-up visit was the main limitation of this study, which may have introduced selection bias and limited the power of the regression analyses.

**Conclusions:**

FM accretion in early life was positively associated with markers of adiposity and lipid metabolism, but not with blood pressure and cardiometabolic markers related to glucose homeostasis. FFM accretion was primarily related to linear growth and FFM at 5 years.

## Abstract 2

Rasmus Wibaek and colleagues reveal associations between fat mass and fat free mass in the first 6 months of life and cardiometabolic markers and growth later in childhood.

## Author summary

**Why was this study done?:**

- Suboptimal growth in fetal life and early childhood are important risk factors for later obesity, diabetes, and heart disease.
- Progression towards disease may start early in life, demonstrated by studies linking early growth with changes in fat mass, lean mass, and cardiometabolic risk markers in children as young as 4 years of age.
- In populations from low-income countries, currently experiencing a rapidly growing chronic disease burden, knowledge about these relationships is lacking.

**What did the researchers do and find?:**

- We followed a cohort of healthy preschool children from the city of Jimma, Ethiopia, for 5 years starting from birth.
- Using advanced statistical growth modelling and accurate assessment of body composition, we estimated individual fat and lean mass accretion in the periods 0–3 and 3–6 months of age in 507 children.
- We conducted regression analyses to test for associations of fat and lean mass growth in early infancy with body composition and cardiometabolic risk markers measured at 5 years of age. These analyses showed that children who gained more fat mass in early infancy had greater fat mass and waist circumference and higher concentrations of risk markers related to cholesterol metabolism at 5 years. Children who gained more lean mass in early infancy were taller and had more lean mass at 5 years.
- Fat and lean mass growth in early infancy were not associated with blood pressure and cardiometabolic markers related to glucose homeostasis at 5 years.

**What do these findings mean?:**

- This study showed that early infancy is an important developmental window for a child’s later body composition.
- This contemporary cohort of preschool children from a sub-Saharan African urban setting had markedly higher fat mass than similar-aged children from the UK and India, which may be a result of excess fat mass accretion during the first 6 months of life.
- Variable patterns of fat and lean mass growth did not appear to be consistently associated with markers of cardiometabolic risk, other than markers of cholesterol metabolism at 5 years.

## Introduction

Non-communicable diseases like type-2-diabetes and cardiovascular diseases are among the leading causes of death and disability worldwide [1,2]. There is mounting evidence that perturbations in fetal and early-life growth increase the risk of a wide range of metabolic disorders such as obesity, type 2 diabetes, and cardiovascular disease in adulthood [3–6]. Over the last 3 decades the research focus has shifted from the consequences of fetal growth restriction, indexed by low birth weight, to the harmful effects of childhood obesity and rapid early growth on a variety of later health outcomes including body size, body composition (BC), and the risk of cardiometabolic diseases. The relative importance of fetal versus postnatal growth has been debated [7,8], but it is likely that both aspects of early growth are involved in the risk development [9,10]. Most studies relating early growth to later health have been conducted in high-income populations. However, as many low- and middle-income countries are currently undergoing rapid nutritional transition and more than 80% of the global mortality burden of non-communicable diseases already occurs in low- and middle-income countries [11], it has become increasingly important to identify critical windows of growth associated with obesity and risk of cardiometabolic diseases in these populations.

Simple anthropometry, such as weight or length, is commonly used to study associations of early growth with later health outcomes. However, as the 2 main components of the growing child, fat mass (FM) and fat-free mass (FFM), have different metabolic properties [12], they most likely contribute differently to the programming of later cardiometabolic disease risk as well as the growth and development in early childhood.

In the Infant Anthropometry and Body Composition (iABC) cohort of more than 500 Ethiopian children with repeated measurements of FM and FFM from birth to 6 months of age, we have previously reported different proportions of FM and FFM among newborns with similar body weights [13], and identified very distinct patterns of FM and FFM growth through infancy [14]. Contrasting accretion patterns in the metabolically diverse tissues of FM and FFM are likely to play a key role in the associations between early growth patterns and later cardiometabolic disease risk. We therefore aimed to study the associations of FM and FFM at birth and their accretion during infancy with height, waist circumference, FM, FFM, and cardiometabolic risk markers at 5 years of age in Ethiopian children.

## Methods

This study is reported as per the Strengthening the Reporting of Observational Studies in Epidemiology (STROBE) guideline (S1 Text). Data for the present study were collected following a prospectively written study protocol (S2 Text).

### Study setting and participants

The iABC study is a prospective birth cohort study of the determinants and consequences of growth variability in early childhood [13,15]. The study was carried out at Jimma University Specialized Hospital in Jimma, Ethiopia (with a population size of 157,432 [16], and situated 350 km southwest of the capital Addis Ababa). Mother–child pairs meeting our eligibility criteria (residing in Jimma, gestational age at birth ≥ 37 completed weeks of pregnancy, birth weight ≥ 1,500 g, no congenital malformations) were enrolled between 17 December 2008 and 24 October 2012. Eligible mother–child pairs were examined within 48 hours of birth and were invited for a total of 12 scheduled visits between birth and 5 years of age. To estimate FM and FFM accretion in infancy, we used data on FM and FFM at birth and at 1.5, 2.5, 3.5, 4.5, and 6 months of age. To capture the dynamics of FM and FFM accretion in early infancy, we required a minimum of 3 assessments of FM and FFM between 0 and 6 months, including an assessment at birth, to be included in the BC growth modelling. The outcome data on BC and cardiometabolic markers were collected at the 5-year visit.

### Data collection

#### Anthropometry and BC in infancy and early childhood

Standing height at 5 years was measured in duplicate to the nearest 0.1 cm (model 213 stadiometer, Seca, Hamburg, Germany). Weight, FM, and FFM from birth to 6 months were assessed with a PEA POD—an infant air displacement plethysmograph (ADP) designed to measure infants between birth and 6 months of age (COSMED, Rome, Italy). At 5 years, weight, FM, and FFM were assessed with a BOD POD—a child/adult ADP with a paediatric chair insert allowing accurate assessment in children above the age of 2 years (COSMED). These ADP instruments provide accurate, precise, feasible, and safe assessment of FM and FFM in infants and children [17–19]. The PEA POD has previously been validated in the iABC cohort against a 3-compartment model of BC incorporating measurement of total body water by stable isotopes [15]. A comprehensive overview of the theory and methods behind the PEA POD and BOD POD techniques is found elsewhere [20,21]. In short, an ADP relies on densitometry to distinguish the 2 body components FM and FFM. First, by measuring total body weight and volume, the total body density is derived. Subsequently, since the density of FM and FFM differs, the relationship between the total body density and the assumed densities of FM and FFM is used to attribute the body weight to either FM or FFM, using a 2-component model of BC and Archimedes’ principle [22]. The density of FM was assumed to be constant at 0.9007 g/cm^3^, while age- and sex-specific densities of FFM were used [23]. The calculations were performed by the inbuilt computers of the PEA POD and the BOD POD, software versions 3.3.0 and 5.2.0, respectively. A complete BC assessment lasted 5–10 minutes, and the 2-minute volume measurement occurred in an enclosed transparent test chamber (PEA POD and BOD POD). In the PEA POD the nude infant was placed in supine position on a tray wearing a swim cap, and in the BOD POD the child sat on a paediatric chair insert wearing a swim cap and tight fitted underpants.

#### Blood pressure at 5 years

After relaxing for a minimum of 5 minutes, systolic and diastolic blood pressure were measured in a sitting position using a blood pressure monitor with age-appropriate cuffs (Pressostabil model, Welch Allyn, Skaneateles Falls, NY, US). Measurements were done in duplicate, and the values averaged.

#### Other cardiometabolic markers at 5 years

After a minimum of 3 hours of fasting, 2 ml of venous blood was drawn from the antecubital fossa. We determined glucose concentrations from whole blood using the HemoCue Glucose 201 RT System (HemoCue, Ängelholm, Sweden). Glycosylated haemoglobin (HbA1c, mmol/mol) was determined from whole blood using a DCCT aligned Quo-Test A1c Analyzer (EKF Diagnostics, Cardiff, Wales). After clotting, the whole blood was centrifuged to isolate serum, divided into three 0.4-ml aliquots and frozen at −80 °C until analysed at the Ethiopian Public Health Institute, Addis Ababa, Ethiopia. Serum concentrations of total cholesterol, low-density lipoprotein (LDL)–cholesterol, high-density lipoprotein (HDL)–cholesterol, and triglycerides (all in mmol/l) were determined using the COBAS 6000, module c501, and insulin (μU/ml) and C-peptide (ng/ml) concentrations were determined using the COBAS 6000, module e601 (Roche Diagnostics International, Rotkreuz, Switzerland). We calculated the homeostasis model assessment of insulin resistance index (HOMA-IR) as insulin × glucose/22.5 [24].

#### Covariates

Maternal postpartum height was measured in duplicate to the nearest 0.1 cm using a Seca 214 stadiometer (Seca, Hamburg, Germany). We used an average of the available measurements from birth to the 6-month visit. Data on birth order of the current child (parity), child’s sex, gestational age at birth, maternal age, maternal educational level, and family socioeconomic status were collected through questionnaires at the birth visit. Gestational age at birth of the current child was assessed using the New Ballard Score test instrument [25]. Socioeconomic status of the family was estimated using the International Wealth Index (IWI). The IWI estimates the wealth status of families in low- and middle-income countries using 12 material well-being items, including 7 items on household assets, 2 items on access to public services, and 3 items on characteristics of the house [26]. The IWI has a range of 0 to 100 (highest wealth). Data on breastfeeding status were collected at the follow-up visits at 4.5 and 6 months after birth and divided into 4 categories: exclusive (no other foods given), almost exclusive (no other foods given except water), predominant (breast milk as primary food), and partial/none (breast milk not the primary food/not breastfeeding) [27]. We used the breastfeeding status at the 6-month visit, but if a child did not attend the 6-month visit we used the breastfeeding status from the visit at 4.5 months of age.

### Ethics

The study was approved by the Ethical Review Committee of Jimma University (Reference RPGC/279/2013). Written, visual, and oral information about the study was presented in local language prior to obtaining written consent from a parent or caregiver. No risks were associated with the examinations, and a topical anaesthetic (EMLA cream) was used prior to collecting the 2 ml of venous blood sample. Medical conditions noticed by the research nurses were addressed according to local clinical guidelines.

### Statistical methods

Descriptive data are presented as mean (standard deviation [SD]) or median (interquartile range) for continuous variables and count (percentage) for categorical variables. Differences between groups were tested by 1-way ANOVA F-test for continuous variables and Pearson’s chi-squared test of independence or Fisher’s exact test of independence for categorical variables. Continuous variables with a right-skewed distribution were log-transformed (natural logarithm) prior to regression analyses. Estimates from these models were back-transformed and presented as percentwise change. A significance level of 5% was used. All analyses were carried out in R version 3.4.1 (R Foundation for Statistical Computing).

#### FM and FFM accretion in early life

Linear-spline mixed-effects (LSME) modelling was used to approximate the non-linear relationship of age with FM and FFM by deriving a number of child-specific and average summary measures of growth over discrete time intervals from 0 to 6 months of age [28,29]. LSME modelling differs from conventional mixed-effects modelling by combining 2 or more linear mixed-effects modelling functions at pre-specified ages (knot points). Thus, the estimated FM and FFM growth velocities are constant within a given time interval but allowed to differ between successive time intervals. Separate LSME models, specified with a knot point at 3 months of age, were fitted for FM and FFM. The LSME models for FM and FFM return 3 average and 3 child-specific growth parameters: estimated FM/FFM at birth and estimated FM/FFM growth velocity in the periods 0–3 and 3–6 months. The child-specific growth parameters are used as continuous exposure variables in the subsequent regression analyses of the BC and cardiometabolic outcomes. A detailed description of the modelling of FM and FFM growth velocity is provided in S3 Text.

#### Associations of FM and FFM accretion in early life with BC and cardiometabolic markers at 5 years

Associations of estimated FM and FFM at birth and their growth velocity over the periods 0–3 months and 3–6 months with cardiometabolic markers and BC at 5 years were analysed in separate multiple regression models (e.g., FM at 5 years regressed on estimated weight gain velocity from 0 to 3 months and adjusted for relevant covariates in separate models). Model 1 was adjusted for child’s sex, birth order, gestational age at birth, child’s exact age at the 5-year visit, maternal age at delivery, maternal postpartum height, maternal educational status, and IWI. Model 2 was additionally adjusted for FM at the 5-year visit. In the regression analyses of FM and waist circumference at 5 years as outcome, model 2 was adjusted for FFM at the 5-year visit instead of FM. We used a complete case approach, limiting the analyses to children with complete data on the included covariates. Covariates included in model 1 and 2 were identified a priori based on adjustment practices in similar studies and reported associations of the covariates with growth in early life and the outcomes studied [30–38]. To obtain comparable estimates across the different growth periods, exposure variables were standardised prior to the regression analyses. Thus, the estimates indicate the change in outcome per study population SD increase of the exposure variable (e.g., FM accretion from 0 to 3 months). We also present estimates for the change in outcome per 100-g higher birth FM and FFM and 100-g/month higher FM and FFM growth velocity over the periods 0–3 and 3–6 months of age. In additional analyses, we accounted for multiple testing using the Benjamini-Hochberg approach [39], with the number of tests set to 90 (15 outcomes and 3 age periods for the 2 exposures FM and FFM). Finally, as data on breastfeeding were only available on a smaller sub-sample, we ran sensitivity analyses on this sample where we adjusted model 1 and 2 for breastfeeding status at 4.5 to 6 months postpartum.

## Results

**Fig 1:**
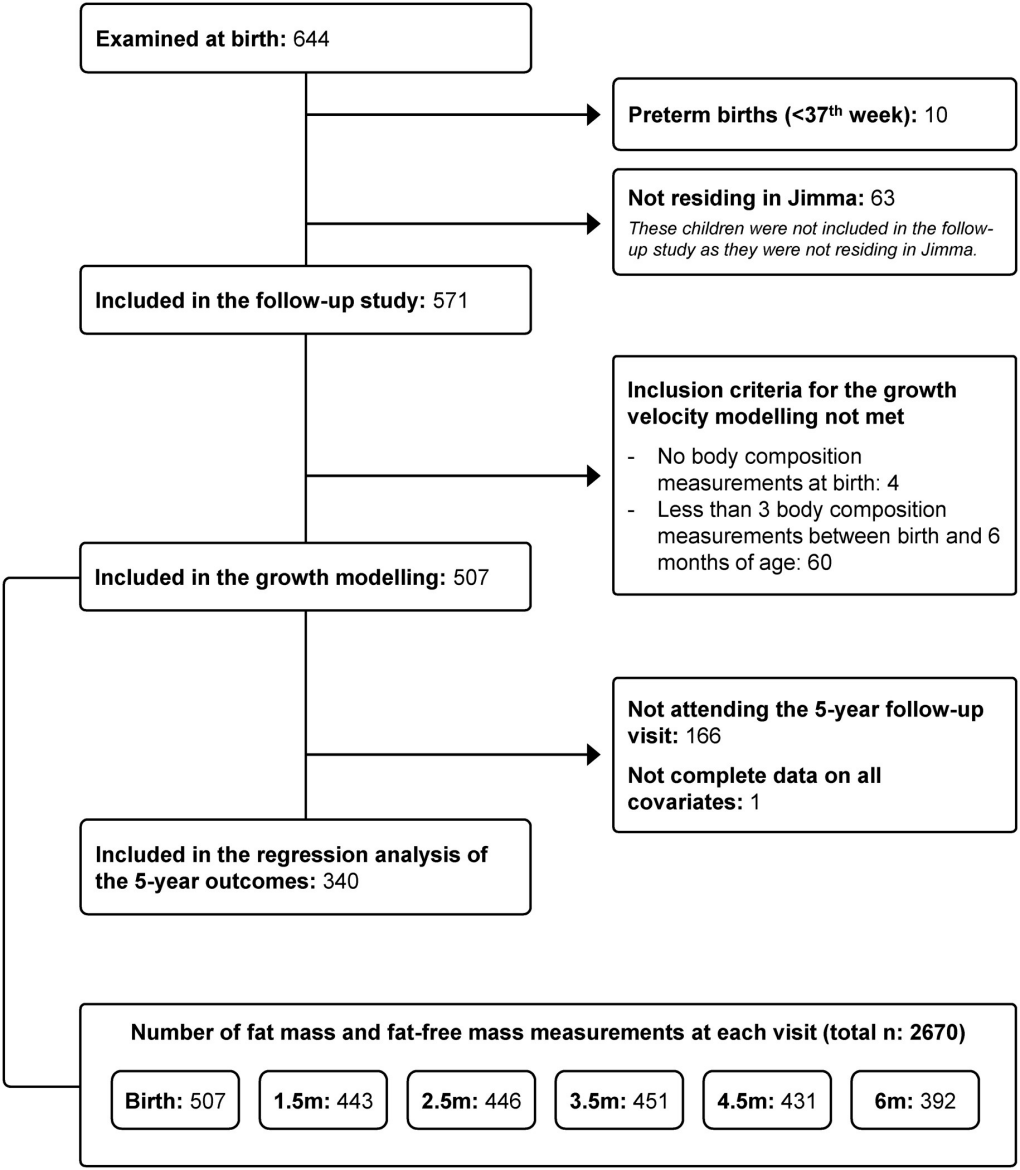
Flow diagram of the study participants and number of fat mass and fat-free mass observations at each follow-up visit from birth to 6 months.

A total of 644 mother–child pairs attended the baseline examination at birth (Fig 1).

Preterm births (*n* = 10) and mother–child pairs not residing in the city of Jimma who had only been to the hospital to give birth (*n* = 63) were excluded. Of the 571 children included in the follow-up study, 507 were eligible to be included in the growth modelling. Of these, 46, 56, 122, and 283 children had their FM and FFM assessed 3, 4, 5, and 6 times, respectively, during the first 6 months of follow-up (median number of measurements: 6). Thus, the LSME growth modelling included a total of 2,670 assessments of FM and FFM. The subsequent regression analyses included 340 children as 166 were not able to attend the 5-year visit or were lost to follow-up and 1 child did not have complete data on all covariates. Background characteristics, BC measures, and cardiometabolic markers of the mother–child pairs included in the regression analyses are presented in Tables 1 and 2.

**Table 1:** Description of the mother–child pairs included in the modelling of fat mass and fat-free mass velocity and attending the 5-year follow-up visit^1^.

Characteristic	Full sample (*n* = 340)	Girls (*n* = 171)	Boys (*n* = 169)	*p*-Value^2^	Missing, *n*
**Maternal characteristics**					
Age at birth (years)	24.5 (4.7), 15.0 to 38.0	24.9 (4.8), 15.0 to 38.0	24.1 (4.5), 16.0 to 37.0	0.117	0
Postpartum height (cm)	157.1 (6.1), 142.0 to 174.0	157.5 (6.3), 143.5 to 174.0	156.6 (5.9), 142.0 to 173.6	0.197	0
Postpartum body mass index (kg/m^2^)	22.20 (3.52), 14.42 to 36.32	22.22 (3.43), 14.42 to 36.32	22.18 (3.61), 15.85 to 34.21	0.919	3
Birth order of current child					
First	168 (49.4)	78 (45.6)	90 (53.3)		
Second	91 (26.8)	45 (26.3)	46 (27.2)		
Third or above	81 (23.8)	48 (28.1)	33 (19.5)	0.162	0
Breastfeeding status at 4.5 to 6 months postpartum					
Exclusive	41 (12.5)	20 (12.0)	21 (13.0)		
Almost exclusive (water given)	70 (21.4)	34 (20.5)	36 (22.4)		
Predominant	198 (60.6)	102 (61.4)	96 (59.6)		
Partial/none	18 (5.5)	10 (6.0)	8 (5.0)	0.938	13
Maternal education					
No school	24 (7.1)	10 (5.8)	14 (8.3)		
Some primary school	153 (45.0)	78 (45.6)	75 (44.4)		
Completed primary school	54 (15.9)	36 (21.1)	18 (10.7)		
Completed secondary school	65 (19.1)	26 (15.2)	39 (23.1)		
Higher education	44 (12.9)	21 (12.3)	23 (13.6)	0.052	0
Socioeconomic status (International Wealth Index)	45.5 (17.1), 8.1 to 93.5	46.2 (17.5), 8.1 to 93.5	44.8 (16.7), 10.4 to 93.5	0.452	0
**Child characteristics at birth**					
Gestational age (weeks)	39.0 (1.0), 37.0 to 42.0	39.1 (1.0), 37.0 to 42.0	39.0 (0.9), 37.0 to 42.0	0.776	0
Weight (kg)	3.05 (0.40), 2.04 to 4.21	3.00 (0.41), 2.05 to 4.21	3.10 (0.40), 2.04 to 4.03	0.027	0
Length (cm)	49.2 (1.9), 43.0 to 54.5	48.9 (1.9), 43.5 to 53.8	49.4 (1.9), 43.0 to 54.5	0.012	0
Fat mass (kg)	0.22 (0.16), −0.17 to 1.06	0.23 (0.16), −0.12 to 0.66	0.21 (0.17), −0.17 to 1.06	0.171	0
Fat-free mass (kg)	2.83 (0.32), 1.95 to 3.80	2.77 (0.31), 1.95 to 3.54	2.89 (0.32), 2.07 to 3.80	<0.001	0
Low birth weight^3^	31 (9.1)	18 (10.5)	13 (7.7)	0.472	0
**Child characteristics at 5 years**					
Age at 5-year visit (months)	59.98 (1.41), 51.29 to 65.12	59.95 (1.58), 51.29 to 65.12	60.01 (1.21), 54.47 to 63.57	0.725	0
Weight (kg)	16.27 (2.06), 11.76 to 26.00	16.15 (2.03), 11.76 to 25.69	16.40 (2.09), 12.52 to 26.00	0.254	0
Height (cm)	104.2 (4.4), 91.5 to 115.0	104.0 (4.3), 93.6 to 114.0	104.3 (4.6), 91.5 to 115.0	0.536	0
Body mass index (kg/m^2^)	14.96 (1.20), 11.47 to 20.89	14.89 (1.30), 11.47 to 20.89	15.03 (1.09), 12.41 to 19.66	0.312	0
Waist circumference (cm)	51.4 (3.0), 43.0 to 62.8	51.1 (3.1), 43.0 to 62.8	51.7 (2.9), 45.2 to 59.0	0.098	1
Weight for age (*z*-score)^4^	−0.90 (0.87), −3.18 to 2.37	−0.90 (0.83), −3.18 to 2.15	−0.90 (0.91), −2.93 to 2.37	0.960	0
Height for age (*z*-score)^4^	−1.17 (0.91), −3.76 to 1.19	−1.12 (0.86), −3.23 to 1.19	−1.22 (0.96), −3.76 to 0.87	0.345	0
BMI for age (*z*-score)^4^	−0.25 (0.87), −3.16 to 2.82	−0.32 (0.90), −3.16 to 2.82	−0.19 (0.83), −2.50 to 2.72	0.152	0
Underweight^5^	32 (9.4)	15 (8.8)	17 (10.1)	0.825	0
Stunted^6^	53 (15.6)	24 (14.0)	29 (17.2)	0.519	0
Wasted by BMI (thinness)^7^	10 (2.9)	8 (4.7)	2 (1.2)	0.104	0
Overweight^8^	14 (4.1)	7 (4.1)	7 (4.1)	1.000	0
Obese^9^	4 (1.2)	2 (1.2)	2 (1.2)	1.000	0

^1^Data are mean (SD), minimum to maximum range, for continuous, normally distributed variables and count (%) for categorical variables.

^2^Differences between girls and boys were calculated by 1-way ANOVA F-test for continuous variables, Pearson’s chi-squared test of independence for categorical variables with expected counts ≥ 5 in all cells, and Fisher’s exact test of independence for categorical variables with expected count in any cell < 5.

^3^Low birth weight is defined as birth weight < 2,500 g.

^4^*z*-Scores are derived using the 2006 (children aged <61 months) and 2007 (children aged ≥61 months) World Health Organization (WHO) child growth standards.

^5^Weight for age more than 2 SD below the sex-specific median of the WHO child growth standards.

^6^Height for age more than 2 SD below the sex-specific median of the WHO child growth standards.

^7^BMI for age more than 2 SD below the sex-specific median of the WHO child growth standards.

^8^BMI for age from 1 to 2 SD above the sex-specific median of the WHO child growth standards [40].

^9^BMI for age more than 2 SD above the sex-specific median of the WHO child growth standards [40].

**Table 2:** Cardiometabolic markers and body composition at 5 years of age in the children included in the modelling of fat mass and fat-free mass velocity and attending the 5-year follow-up visit^1^.

Outcome	Full sample (*n* = 340)	Girls (*n* = 171)	Boys (*n* = 169)	*p*-Value^2^	Missing, *n*
**Glucose homeostasis**					
Whole blood glucose (mmol/l)	5.89 (0.84), 3.80–9.90	5.86 (0.76), 3.80–8.40	5.93 (0.91), 4.10–9.90	0.444	23
HbA1c (mmol/mol)	38 (4), 27–62	38 (4), 29–55	37 (4), 27–62	0.479	79
Insulin (μU/ml)^3^	5.98 (3.20–11.20), 0.20–45.99	7.24 (4.06–12.83), 0.20–45.99	5.35 (2.96–9.38), 0.20–33.33	0.005	31
C-peptide (ng/ml)^3^	1.06 (0.65–1.51), 0.14–5.56	1.14 (0.75–1.69), 0.21–5.56	0.92 (0.56–1.41), 0.14–4.46	0.003	36
HOMA-IR^3^^,^^4^	1.29 (0.66–2.47), 0.03–12.21	1.56 (0.85–2.66), 0.03–12.21	1.11 (0.61–2.09), 0.05–8.32	0.008	31
**Lipids**					
Total cholesterol (mmol/l)	3.41 (0.61), 2.04–5.83	3.45 (0.64), 2.04–5.83	3.38 (0.58), 2.19–5.16	0.345	27
LDL cholesterol (mmol/l)	1.65 (0.56), 0.00–3.72	1.69 (0.58), 0.33–3.72	1.61 (0.55), 0.00–3.40	0.180	28
HDL cholesterol (mmol/l)	0.79 (0.26), 0.09–1.53	0.78 (0.27), 0.11–1.53	0.80 (0.24), 0.09–1.50	0.403	32
Triglycerides(mmol/l)^3^	0.95 (0.73–1.28), 0.38–3.75	0.93 (0.76–1.28), 0.44–3.75	0.97 (0.71–1.28), 0.38–3.52	0.721	32
**Blood pressure**					
Systolic (mm Hg)	87.6 (7.2), 70.0–110.0	87.9 (7.1), 70.0–110.0	87.3 (7.4), 70.0–110.0	0.446	2
Diastolic (mm Hg)	54.2 (8.5), 40.0–80.0	54.5 (8.4), 40.0–80.0	54.0 (8.5), 40.0–70.0	0.547	2
**Body composition**					
Fat mass (kg)	4.14 (1.27), 1.17–9.83	4.11 (1.37), 1.17–9.83	4.16 (1.17), 1.48–8.41	0.715	16
Fat-free mass (kg)	12.14 (1.40), 9.29–17.99	12.02 (1.30), 9.34–17.73	12.26 (1.50), 9.29–17.99	0.119	16
Fat mass index (kg/m^2^)	3.80 (1.07), 1.12–7.99	3.79 (1.17), 1.12–7.99	3.80 (0.96), 1.45–6.36	0.881	16
Fat-free mass index (kg/m^2^)	11.18 (0.86), 8.96–13.96	11.12 (0.88), 8.96–13.64	11.25 (0.84), 9.39–13.96	0.187	16

^1^Data are mean (SD), minimum–maximum range, for continuous variables that are normally distributed and median (interquartile range), minimum–maximum range, for continuous variables that do not follow a normal distribution.

^2^Differences between groups were calculated by 1-way ANOVA F-test for continuous, normally distributed variables. Variables found not to follow a normal distribution were log-transformed prior to the tests of group differences.

^3^Non-normally distributed.

^4^Homeostasis model assessment of insulin resistance (HOMA-IR) was calculated as insulin (μU/ml) × glucose (mmol/l)/22.5.

The vast majority of mothers were either exclusively or predominantly breastfeeding at 4.5 to 6 months postpartum, and 48% had completed primary school or a higher level of education. The average wealth status of the families was slightly lower than the national urban average, but considerably higher than the national rural average (46/100 versus 52/100 [urban] and 12/100 [rural]) [26,41]. At birth, 9% were born with low birth weight, which is lower than the average for sub-Saharan Africa of 14% [42]. Compared to the WHO international growth standards [43], at 5 years, the children had on average a lower weight, height, and BMI for age. Stunting at 5 years was seen in 15.5% of the children, which is similar to the proportion in the capital, Addis Ababa, but markedly lower than the national urban average of 25% [44].

### FM and FFM accretion in early infancy

**Fig 2:**
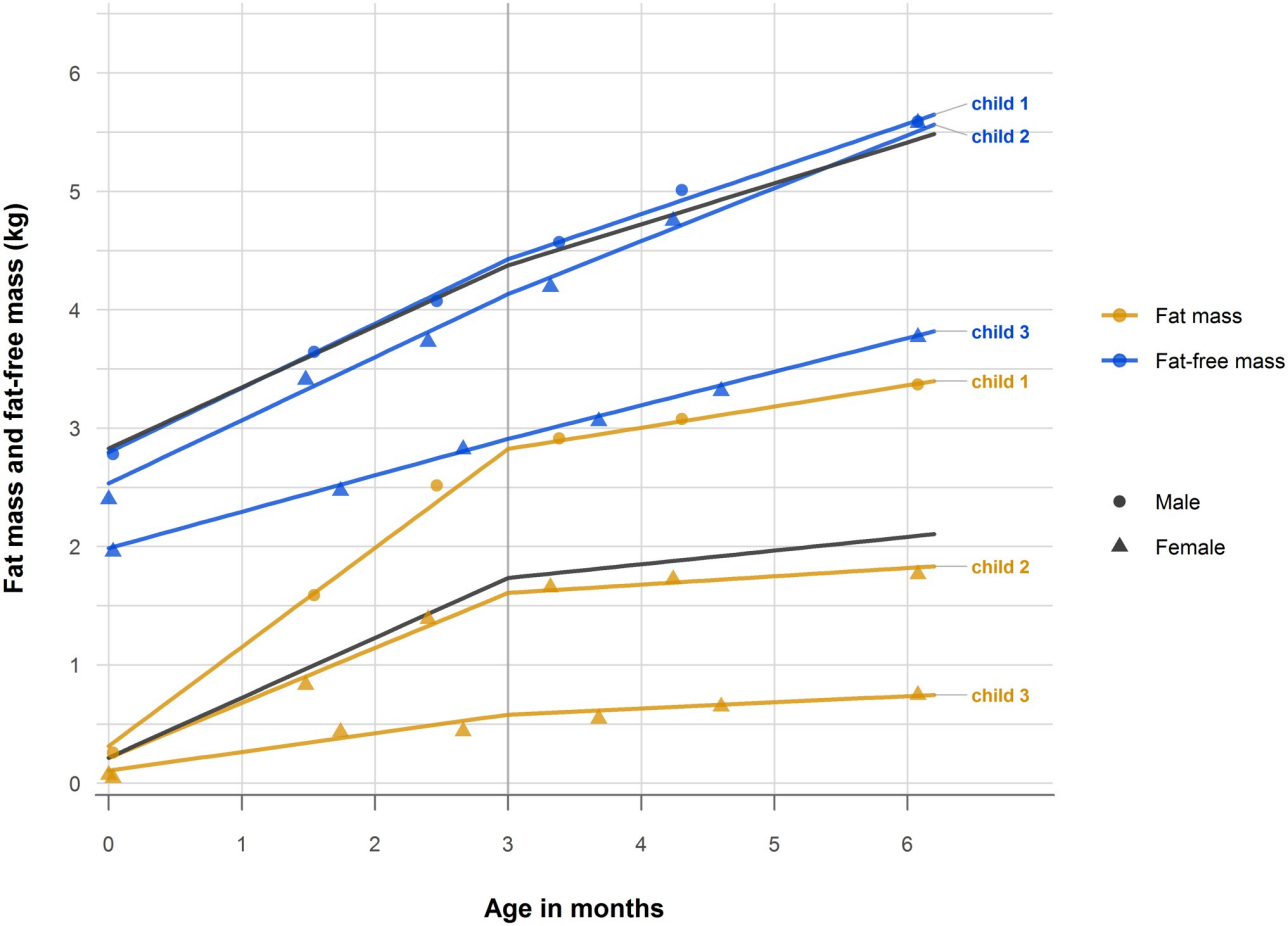
Growth velocity modelling of fat mass and fat-free mass. Estimated child-specific fat mass and fat-free mass growth velocity curves for 3 selected children (coloured curves) and average fat mass and fat-free mass growth velocity curves for the whole study sample (black curves), estimated from linear-spline mixed-effects modelling. The coloured points show the observed fat mass and fat-free mass measurements for each child. The vertical grey line shows the selected knot point at 3 months, and the slopes on each side of the knot point are the estimated growth velocities for each child. The graphs show how the estimated fat mass and fat-free mass at birth (intercept) and growth velocities (the slope parameters for fat mass and fat-free mass) may vary between children.

The LSME models estimated FM and FFM at birth and FM and FFM growth velocity over the periods 0–3 and 3–6 months. Examples of estimated growth velocity curves for 3 selected children are presented in Fig 2.

During the first 6 months, the children had a much larger variation in FM growth velocity compared to FFM growth velocity (S1 and S2 Figs). The FM growth velocity from 0 to 3 months was on average 4.4 times greater than that from 3 to 6 months. Although the estimated FM was only 8% of the FFM component at birth, the average FM growth velocity from 0 to 3 months was similar to that of FFM of 0.5 kg per month. This resulted in a considerable relative catch-up in FM compared to FFM in the first 3 months, and at 6 months the estimated FM was on average only 2.6 times smaller than FFM (Table 3).

**Table 3:** Average estimated fat mass and fat-free mass at birth, fat mass and fat-free mass velocities from birth to 6 months of age estimated with linear-spline mixed-effects modelling, and estimated fat mass and fat-free mass at 6 months in Ethiopian children^1^.

Body composition	Full sample (*n* = 507)	Girls (*n* = 250)	Boys (*n* = 257)	*p*-Value^2^	Fomon’s reference child^3^	
					Girls	Boys
Fat mass						
Estimated fat mass at birth (g)	217 (60)	213 (62)	220 (58)	0.156	495	486
Fat mass velocity, 0–3 months (g/month)	506 (138)	503 (136)	509 (140)	0.639	290	336
Fat mass velocity, 3–6 months (g/month)	116 (104)	122 (103)	109 (105)	0.162	183	181
Estimated fat mass at 6 months (g)	2,081 (613)	2,088 (595)	2,074 (631)	0.799	1,915	2,037
Observed fat mass at 60 months (g)^4^	4,142 (1,275)	4,113 (1,369)	4,172 (1,172)	0.673	2,949	2,720
Fat-free mass						
Estimated fat-free mass at birth (g)	2,828 (286)	2,770 (290)	2,885 (271)	<0.001	2,830	3,059
Fat-free mass velocity, 0–3 months (g/month)	516 (77)	491 (67)	540 (79)	<0.001	516	627
Fat-free mass velocity, 3–6 months (g/month)	346 (68)	336 (65)	357 (70)	0.001	319	351
Estimated fat-free mass at 6 months (g)	5,414 (540)	5,250 (511)	5,573 (519)	<0.001	5,335	5,993
Observed fat-free mass at 60 months (g)^4^	12,137 (1,403)	12,021 (1,302)	12,258 (1,496)	0.129	14,711	15,950

^1^Data are mean (SD).

^2^Differences between the Ethiopian boys and girls were calculated by 1-way ANOVA F-test.

^3^Fat mass and fat-free mass velocity (g/month) were calculated by the difference in mean fat mass and fat-free mass in grams at the end of the age interval and at the beginning of the age interval divided by the length in months of the age interval using data from the 1982 Fomon reference child [23].

^4^*n* for full sample = 325.

Correlations between FM at birth and FM growth velocities as well as FFM at birth and FFM growth velocities are shown in pairs plots in S3 Fig. Matrices of the model assumption tests of the LSME modelling of FM and FFM growth velocity are shown in S4 Fig.

### Associations of FM and FFM accretion in infancy with BC and cardiometabolic markers at 5 years

**Fig 3:**
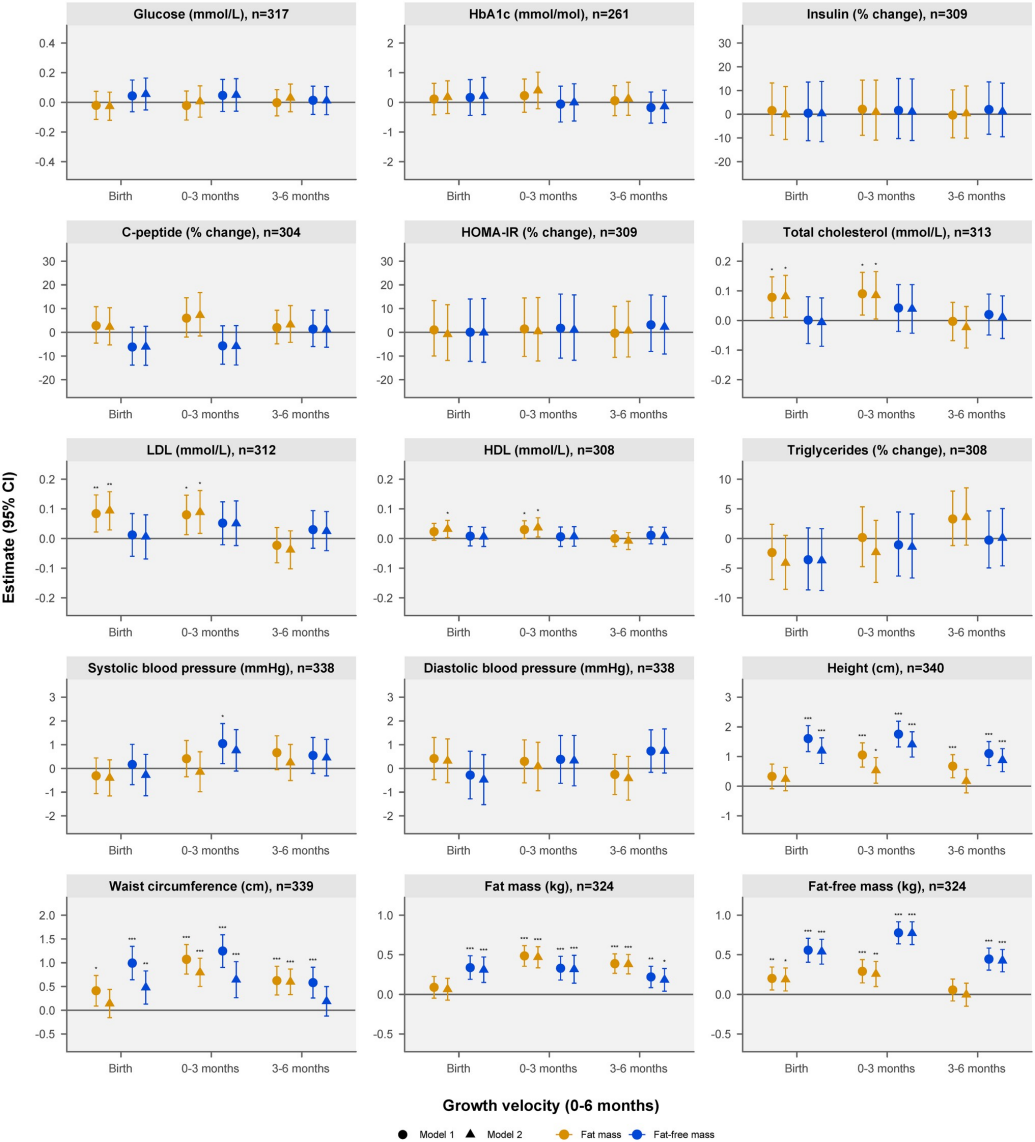
Associations of estimated fat mass and fat-free mass at birth and fat mass and fat-free mass growth velocity over the periods 0–3 months and 3–6 months with body composition and cardiometabolic markers at 5 years. The coefficients (and 95% CIs) displayed in the forest plots were derived from separate multiple linear regression analyses and represent the change in the 5-year outcomes per study population standard deviation increase of estimated fat mass and fat-free mass at birth and fat mass and fat-free mass growth velocity over the periods 0–3 months and 3–6 months. The linear-spline mixed-effects model used to derive the estimated values at birth and growth velocities had 1 knot point at 3 months, yielding the 2 growth periods 0–3 and 3–6 months. Variables found not to follow a normal distribution (i.e., insulin, C-peptide, HOMA-IR, and triglycerides) were log-transformed prior to the regression analyses. The presented estimates for these variables were back-transformed and are shown as percentwise change. Model 1 was adjusted for child’s sex, birth order, gestational age at birth, child’s exact age at the 5-year visit, maternal age at delivery, maternal postpartum height, maternal educational status, and family socioeconomic status (International Wealth Index). Model 2 was additionally adjusted for fat mass at the 5-year visit (applies to all outcomes except fat mass and waist circumference, which were adjusted for fat-free mass at the 5-year visit instead of fat mass in model 2). *p < 0.05, **p < 0.01, ***p < 0.001.

Associations of estimated FM and FFM at birth and FM and FFM growth velocity in the periods 0–3 and 3–6 months with BC and cardiometabolic markers at 5 years are presented in Fig 3. Tabular presentations of the results of the regression analyses are shown in S1 and S2 Tables.

Higher FFM, but not FM, at birth and higher FM and FFM accretion in the periods 0–3 and 3–6 months were associated with higher FM at 5 years. For instance, independent of current FFM and other covariates, 100-g/month higher FM accretion in the periods 0–3 and 3–6 months was associated with 339 g (95% CI: 243–435 g, *p <* 0.001) and 367 g (95% CI: 250–484 g, *p <* 0.001) greater FM at 5 years, respectively (S2 Table). Higher FM and FFM at birth and accretion from 0–3 months were associated with higher FFM at 5 years. FFM accretion from 3 to 6 months was also associated with FFM at 5 years. For instance, independent of current FM and the other covariates, 100-g higher FFM at birth was associated with 189 g (95% CI: 134–243 g, *p <* 0.001) greater FFM, and in the periods 0–3 and 3–6 months, 100-g/month higher FFM accretion was associated with 1,002 g (95% CI: 815–1,189 g, *p <* 0.001) and 624 g (95% CI: 419–829 g, *p <* 0.001) greater FFM at 5 years, respectively. Additionally, FFM at birth and accretion in the periods 0–3 and 3–6 months of age as well as FM accretion in the period 0–3 months were positively associated with height at 5 years. For instance, 100-g/month higher FFM accretion in the period 0–3 months was associated with 1.8 cm (95% CI: 1.3–2.4 cm, *p <* 0.001) greater height at 5 years, and 100-g/month higher FM accretion in the same period with 0.4 cm (95% CI: 0.1–0.7, *p* = 0.016) greater height. FM velocity in the periods 0–3 and 3–6 months, and FFM at birth and velocity from 0–3 months, were positively associated with waist circumference at 5 years.

Higher FM at birth and accretion from 0 to 3 months was associated with higher concentrations of total, LDL-, and HDL-cholesterol, and associations were strongest for LDL-cholesterol. For instance, independent of current FM and the other covariates, 100-g higher FM at birth was associated with 0.16 mmol/l (95% CI: 0.05–0.26 mmol/l, *p* = 0.005) greater LDL-cholesterol at 5 years, and 100-g/month greater FM accretion in the period 0–3 months with 0.06 mmol/l (95% CI: 0.01–0.12 mmol/l, *p* = 0.016) greater LDL-cholesterol (S2 Table). Neither FFM at birth nor accretion over the periods 0–3 and 3–6 months was associated with cholesterol concentrations at 5 years.

In general, the reported associations did not change markedly from model 1 to model 2. The presented associations also did not change markedly after adjusting for breastfeeding status at 4.5 to 6 months postpartum (S5 Fig). When accounting for multiple testing in model 2, the associations for the markers of lipid metabolism were no longer significant except for the association of FM at birth with LDL-cholesterol at 5 years (S6 Fig). The associations of FM and FFM at birth and their accretion in infancy with height, waist circumference, FM, and FFM largely remained significant in the model 2 analyses. We found no associations of FM and FFM at birth and their accretion in infancy with any of the other studied cardiometabolic markers including glucose, HbA1c, insulin, C-peptide, HOMA-IR, triglycerides, and systolic and diastolic blood pressure.

## Discussion

In this contemporary cohort of urban Ethiopian children, we found that FM accretion in infancy was positively associated with markers of adiposity (FM and waist circumference) at 5 years, and that higher FFM at birth and accretion in infancy were associated with FFM, FM, height, and waist circumference at 5 years. In addition, we found that higher FM accretion during fetal life, indexed by FM at birth, and higher FM accretion from 0 to 3 months predicted higher cholesterol concentrations at 5 years, with the strongest association for LDL-cholesterol. However, none of the other studied cardiometabolic markers were associated with FM or FFM at birth or their accretion during infancy.

In high-income populations, fetal and postnatal growth have consistently been related to indices of obesity and markers of cardiometabolic disease risk [5,6,45]. These findings have been crucial for the identification of critical windows in early life in which later obesity and cardiometabolic disease risk are being programmed. However, previous studies have treated the body as a single component by using weight or BMI as the index of growth, and have therefore not been able to assess how the metabolically diverse body components of FM and FFM might mediate associations of growth in early life with later BC and cardiometabolic risk markers. There is also less information on such associations in low- and middle-income populations, though data from India support the notion that fetal life and infancy are key critical windows [34,46,47]. We have previously shown that greater FM accretion from 0 to 4 months predicted higher FM at age 4 years, and that greater FFM accretion from 0 to 6 months predicted higher FM and FFM at 4 years of age [32]. To our knowledge, no other cohort studies have examined the associations of directly assessed FM and FFM accretion during infancy with BC and cardiometabolic risk markers in early childhood. Using a 3-way conditional growth analysis of weight, height, and skinfolds to approximate fat and lean tissue gain in discrete age intervals from birth to 13.5 years, Krishnaveni et al. [33] found that faster fat accretion from 5 to 9.5 years predicted higher fat percentage, waist–hip ratio, systolic blood pressure, insulin, and HOMA-IR at 13.5 years, but they reported no associations of fat or lean tissue at birth and growth over the periods 0–1 and 1–2 years with later BC or any of the studied cardiometabolic outcomes. In a small observational study, Koontz et al. [48] found that accelerated FM accretion from 0 to 8 months of age was associated with an 8-fold increase in the odds of overweight/obesity at 9 years. Previous studies have used changes in weight and/or height as indicators of growth and have found birth weight and weight gain in infancy to be associated with later FM and overweight/obesity [48–50], FFM [37,51], or both FM and FFM [52,53].

The mechanisms relating higher FFM accretion in infancy to higher FFM in childhood and higher FM accretion in infancy to higher FM in childhood remain elusive. Besides cellular and extracellular water, FFM consists primarily of protein followed by osseous minerals [23]. In a large birth cohort study from the Netherlands, higher fetal and postnatal growth in weight and height predicted higher bone mineral content, density, and area [54]. The strongest associations for the bone outcomes were seen for growth in the first year. Greater FFM accretion in early infancy may therefore promote bone growth, likely through a complex interplay with growth hormone and insulin-like growth factor 1 (IGF-1) [55], which in turn contributes to higher FFM later. In addition, the accretion of muscle mass is likely to be a beneficial investment for later cardiometabolic health. Even though FFM accretion was not related to markers of glucose homeostasis at 5 years in the present study, muscle is an important factor for the uptake of glucose from the blood in response to insulin secretion, and low FFM may therefore influence later insulin sensitivity [51].

Regarding the cardiometabolic risk markers, we found that FM at birth and accretion from 0 to 3 months were positively associated with cholesterol concentrations at 5 years, with the largest estimates for LDL-cholesterol. Interestingly, the associations remained after controlling for current FM. While FM is important for brain development and resilience to infections in early life [56], excessive FM accretion in infancy may also lead to harmful levels of FM and a perturbed lipid metabolism later in childhood. As shown in Table 3, compared to reference data for child BC developed by Fomon et al. [23], both boys and girls in the present study had less than half as much absolute FM at birth. At 6 months of age, absolute FM was slightly higher for the Ethiopian boys and around 175 g higher for girls. Surprisingly, relative to the Fomon reference values, the Ethiopian boys and girls had over 1.4 and 1.2 kg higher FM, but a deficit in FFM of 3.7 and 2.7 kg, respectively, at 5 years of age. Additionally, compared to BC reference data from the UK, the Ethiopian children also had higher FM (boys: 1.1 kg higher; girls: 0.2 kg higher) and markedly lower FFM (boys: 4.1 kg lower; girls: 2.6 kg lower) at 5 years [57]. In fact, even compared to 6-year-old children from Pune in India [34], already at 5 years of age, the Ethiopian children had a higher absolute FM (boys: 1.3 kg higher; girls: 0.8 kg higher) and lower absolute FFM (boys: 0.7 kg lower; girls: 0.1 kg lower). The Indian children have been described as having a ‘thin–fat’ phenotype, because of their low weight at birth, short stature, and relatively high FM at birth and in childhood, and have been recognised as being particularly vulnerable to developing obesity and cardiometabolic diseases later in life [58]. In this Ethiopian population, we see an even more pronounced thin–fat phenotype at 5 years, resulting from a considerable catch-up in FM, and this may explain some of the variation we see in the cholesterol concentrations at 5 years. Only a few children presented lipid concentrations exceeding the cutoffs for high total and LDL-cholesterol [59], and at this age we cannot say whether increases in total and LDL-cholesterol associated with FM accretion in infancy have health implication in the longer term or rather represent beneficial metabolic adaptations to greater tissue mass. However, cardiometabolic risk markers have been found to track from childhood to adulthood [60,61], and adiposity in childhood has been associated with risk of obesity and cardiometabolic disease later in life [62–64]. Furthermore, even small increases in LDL-cholesterol could potentially promote atherosclerotic processes already in childhood, as the probability of intimal retention of LDL-cholesterol particles, and hence the risk of developing atherosclerotic plaques, increases in a dose–response relationship when circulating concentrations of LDL-cholesterol exceed concentrations as low as 0.5–1.0 mmol/l [65]. Furthermore, the rate of atherosclerotic plaque formation is further increased with risk factors such as genetic predisposition, high blood pressure, high-fat diets, and adiposity [66]. On average, the HDL-cholesterol concentration was 0.79 mmol/l, which is well below the recommendation for children that HDL-cholesterol concentration should be above 1.16 mmol/l [59]. Thus, a low average birth weight, a high level of FM, a low level of FFM, and a low concentration of HDL-cholesterol at school age as well as exposure to a rapidly changing obesogenic food environment could potentially interact with LDL-cholesterol concentrations and make Ethiopian children and similar populations particularly vulnerable to later cardiometabolic disease. Surprisingly, we also found that FM at birth and accretion from 0 to 3 months were associated with greater HDL-cholesterol, which could suggest general high turnover of apolipoproteins with adipose tissue accumulation.

### Strengths and limitations

A major strength of this study was the detailed assessment of FM and FFM from birth to 6 months and again at 60 months, using accurate and validated equipment for estimation of BC. A high density of BC data during the first 6 months allowed us to robustly estimate the non-linear relationship of FM and FFM as a function of age in critical windows of development in early childhood using LSME modelling. This modelling approach provided easily interpretable growth coefficients from a complex data structure of repeated measures, and at the same time accounted for the dependencies of the child-specific BC measurements. Our growth models also allowed children to be included in the modelling even if they were not measured at the exact same time or had not attended all 6 follow-up visits during infancy. Thus, as opposed to more conventional growth modelling approaches, such as conditional growth modelling or tracing average *z*-scores, that require a complete case analysis, we were able to reduce potential bias from selective dropout and increase the statistical power of the study considerably. This novel approach to growth modelling has been implemented successfully in previous studies [28,67,68]. Finally, of the children initially included in the follow-up study (*n* = 571), 89% were included in the modelling of FM and FFM accretion in infancy, and 60% of the mother–child pairs were able to participate in the follow-up visit at 5 years, where data on the cardiometabolic and BC outcomes were collected.

Among the limitations, we cannot rule out that the loss of mother–child pairs examined at birth but not able to attend the visit at 5 years could have introduced selection bias, although mother–child pairs lost to follow-up were generally similar to those included in the 5-year analyses (S3 Table). Also, as the PEA POD was only able to measure children from birth to 6 months of age, we did not have FM and FFM assessments in the period from 6 months to 4 years. Previous studies have found that weight gain in early childhood, rather than infancy and fetal life, is the strongest correlate of concentrations of insulin and C-peptide and of HOMA-IR [31,34]. Thus, information on FM and FFM accretion from 6 months to 4 years could have provided valuable insight into the relative importance of FM and FFM accretion beyond infancy. Additionally, as it was not feasible for the small children to undergo an overnight fast, the fasting time was standardised to 3 hours. This may have caused some non-differential misclassification of the estimates related to glucose homeostasis (i.e., estimates moving towards 0). However, for the lipid measurements, as most children are in a non-fasting state most of the day, the concentrations observed after 3 hours of fasting are likely to be a better reflection of the lipid and lipoprotein concentrations in the circulating blood than after an overnight fast [69]. The advantages of using non-fasting measurements of daily lipid and lipoprotein concentrations have resulted in recommendations to include non-fasting lipid measurements in several national society guidelines, including the Danish Society of Clinical Biochemistry and the National Institute for Health and Care Excellence (NICE) in the UK. Finally, the observational study design precludes us from making any claims of causal inference, and we can therefore not exclude the possibility that residual confounding from important covariates such as pre-pregnancy maternal nutritional status, gestational weight gain, paternal BMI, fetal growth trajectories, diet in infancy and childhood, and duration of breastfeeding could have resulted in the identified associations. A longer duration of breastfeeding is inversely associated with the risk of overweight [70]. Moreover, mode of infant feeding is associated with differences in early-life accretion of FM and FFM, with lower FM accretion from birth to 6 months among formula-fed compared to breastfed infants but higher FM at 12 months [71]. Correspondingly, in the present cohort, we have previously shown that not being breastfed at 3 months of age was associated with a delayed FM accretion pattern [14]. Evidence on the cardiometabolic effects of breastfeeding remains controversial, with some studies suggesting a beneficial effect of breastfeeding in reducing adiposity and cardiometabolic risk [72–74] and others finding no to little positive effect on various cardiometabolic outcomes [75–78]. Our breastfeeding data were crude, and unfortunately we did not have data on complementary feeding. Nevertheless, in a sensitivity analysis, we adjusted our main analysis for breastfeeding status at 4.5 to 6 months postpartum, which did not affect the identified associations markedly (S5 Fig). Furthermore, the high levels of predominant and exclusive breastfeeding in this population suggest that breastfeeding may not have been the main source of variability.

Our findings suggest that development of adiposity and changes in cardiometabolic risk markers related to lipid metabolism may occur already in early life and are likely to be driven by accelerated fetal and early postnatal FM accretion. In contrast, FFM accretion in early life is likely to improve cardiometabolic status through linear growth and continued accretion of lean tissue, even though we were not yet able to demonstrate any direct benefits in the cardiometabolic markers. Thus, based on existing evidence from the iABC cohort and the present findings, we stress the importance of promoting lean tissue accretion and linear growth without excess accumulation of adipose tissue in countries undergoing rapid economic and nutritional transitions, as children in such contexts are likely to be particularly exposed to an environment conducive to an unhealthy lifestyle. This may be done through public health policies that promote healthy diets and physical activity in preschools and schools as well as limit the exposure to obesogenic environments (outlets with soft drinks, fast food, and unhealthy snacks) within schools and in the community [79]. Lastly, promotion of exclusive breastfeeding as the most optimal nutrition for the child is key to ensure normal growth, prevent infections, and reduce the risk obesity in childhood and adolescence [78].

## Supporting information

S1 Fig Growth velocities from birth to 6 months of age for fat mass and fat-free mass estimated with linear-spline mixed-effects modelling. (PDF)

S2 Fig Density plots of the variation of fat mass and fat-free mass growth velocity in the periods 0–3 and 3–6 months of age. (PDF)

S3 Fig Correlation matrix (pairs plot) of the child-specific standard deviation (SD) scores of estimated fat mass and fat-free mass at birth and SD scores of fat mass and fat-free mass growth velocities in the periods 0–3 and 3–6 months. (PDF)

S4 Fig Matrix of model assumption tests of the linear-spline mixed-effects modelling of fat mass and fat-free mass growth velocity. (PDF)

S5 Fig Sensitivity analyses of the associations shown in Fig 3. Associations of estimated fat mass and fat-free mass at birth and fat mass and fat-free mass growth velocity in the periods 0–3 and 3–6 months with body composition and cardiometabolic risk markers at 5 years (all analyses adjusted for breastfeeding at 4.5 to 6 months postpartum).
(PDF)

S6 Fig Sensitivity analyses accounting for multiple testing in model 2 of the associations of estimated fat mass and fat-free mass at birth and fat mass and fat-free mass growth velocity for the periods 0–3 and 3–6 months with body composition and cardiometabolic risk markers at 5 years. (PDF)

S1 Table Associations of predicted fat mass and fat-free mass at birth and fat mass and fat-free mass growth velocity in the periods 0–3 and 3–6 months with cardiometabolic markers and body composition at 5 years in the fully adjusted model 2 (exposures in SD units). (PDF)

S2 Table Associations of predicted fat mass and fat-free mass at birth and fat mass and fat-free mass growth velocity in the periods 0–3 and 3–6 months with cardiometabolic markers and body composition at 5 years in the fully adjusted model 2 (exposures in absolute values). (PDF)

S3 Table Comparison of background characteristics of the mother–child pairs attending the 5-year follow-up visit with those not attending. (PDF)

S1 Text STROBE statement. (PDF)

S2 Text Study protocol: Body composition trajectories and metabolic risk in African children. (PDF)

S3 Text Detailed description of the linear-spline mixed-effects modelling. (PDF)

## Abbreviations

ADP: air displacement plethysmograph
BC: body composition
FFM: fat-free mass
FM: fat mass
HDL: high-density lipoprotein
iABC: Infant Anthropometry and Body Composition
IWI: International Wealth Index
LDL: low-density lipoprotein
LSME: linear-spline mixed-effects
