# Supplementary material for: Associations of fat mass and fat-free mass accretion in infancy with body composition and cardiometabolic risk markers at 5 years: The Ethiopian iABC birth cohort study
Source: PLoS Med. 2019 Aug 20;16(8):e1002888. doi: 10.1371/journal.pmed.1002888 (PMC6701744; doi:10.1371/journal.pmed.1002888)

S1 Fig. Growth velocities from birth to 6 months of age for fat mass and fat-free mass estimated with linear-spline mixed-effects modelling. The grey curves show the estimated growth velocities for each child and the coloured curve the average growth velocities for the whole study population in the periods 0-3 and 3-6 months of age. The vertical grey line shows the selected internal knot point at 3 months, and the slopes on each side of the knot point are the growth velocities for each child.

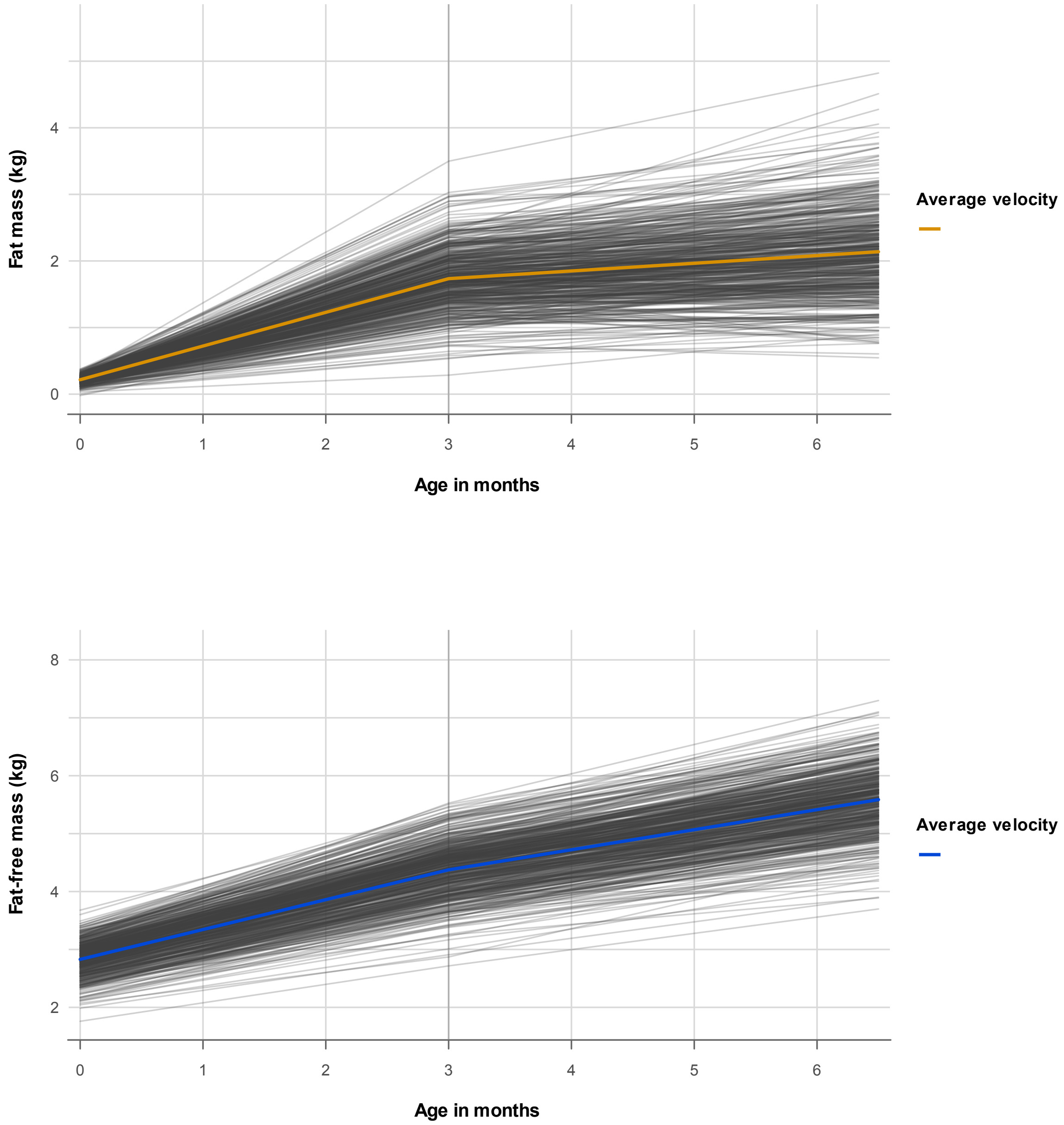

Supplement: S1 Fig — (PDF) [file pmed.1002888.s001.pdf]
