# Supplementary material for: Associations of fat mass and fat-free mass accretion in infancy with body composition and cardiometabolic risk markers at 5 years: The Ethiopian iABC birth cohort study
Source: PLoS Med. 2019 Aug 20;16(8):e1002888. doi: 10.1371/journal.pmed.1002888 (PMC6701744; doi:10.1371/journal.pmed.1002888)

S2 Fig. Density plots of the variation of fat mass and fat-free mass growth velocity in the periods 0-3 and 3-6 months of age. The distributions are based on a kernel density estimation. The grey vertical lines show the mean growth velocity in each period.

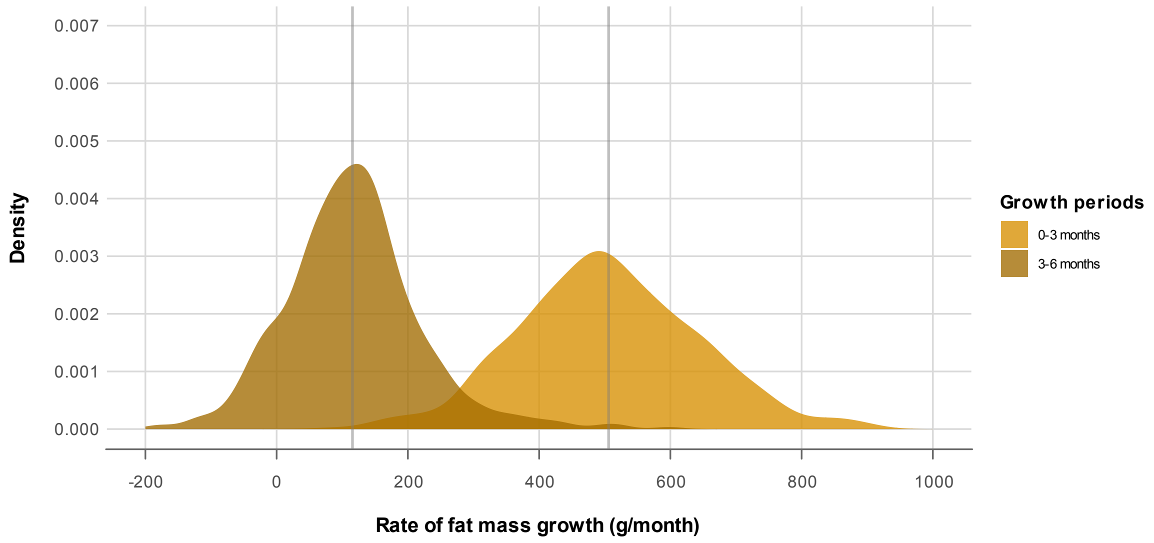

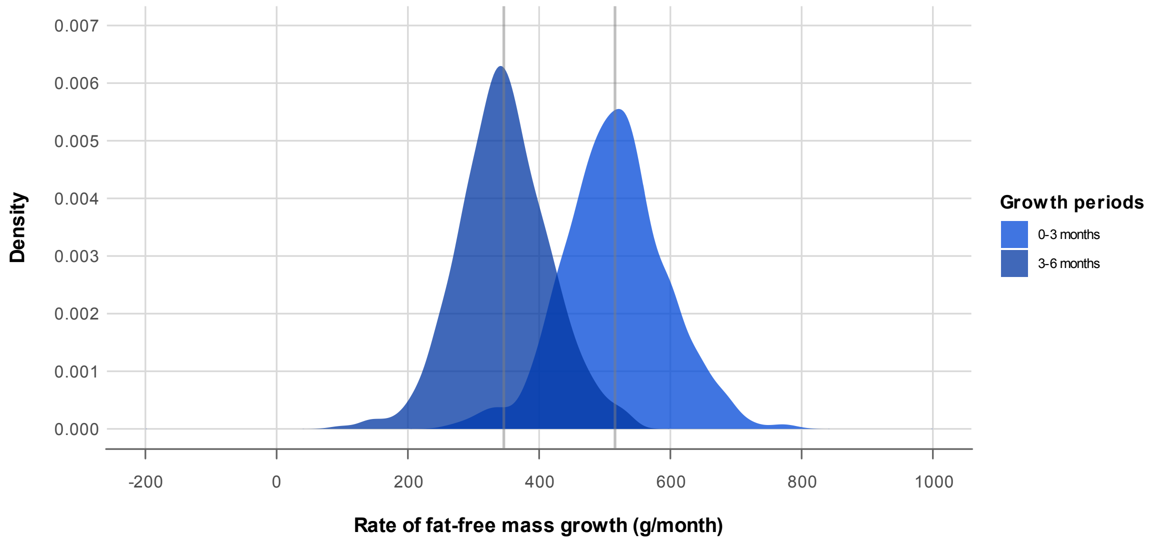

Supplement: S2 Fig — (PDF) [file pmed.1002888.s002.pdf]
