# Supplementary material for: Associations of fat mass and fat-free mass accretion in infancy with body composition and cardiometabolic risk markers at 5 years: The Ethiopian iABC birth cohort study
Source: PLoS Med. 2019 Aug 20;16(8):e1002888. doi: 10.1371/journal.pmed.1002888 (PMC6701744; doi:10.1371/journal.pmed.1002888)
Supplement: S3 Fig — (PDF) [file pmed.1002888.s003.pdf]

S3 Fig. Correlation matrix (pairs plot) of the child-specific standard deviation (SD) scores of estimated fat mass and fat-free mass at birth and SD scores of fat mass and fat-free mass growth velocities in the periods 0-3 and 3-6 months.

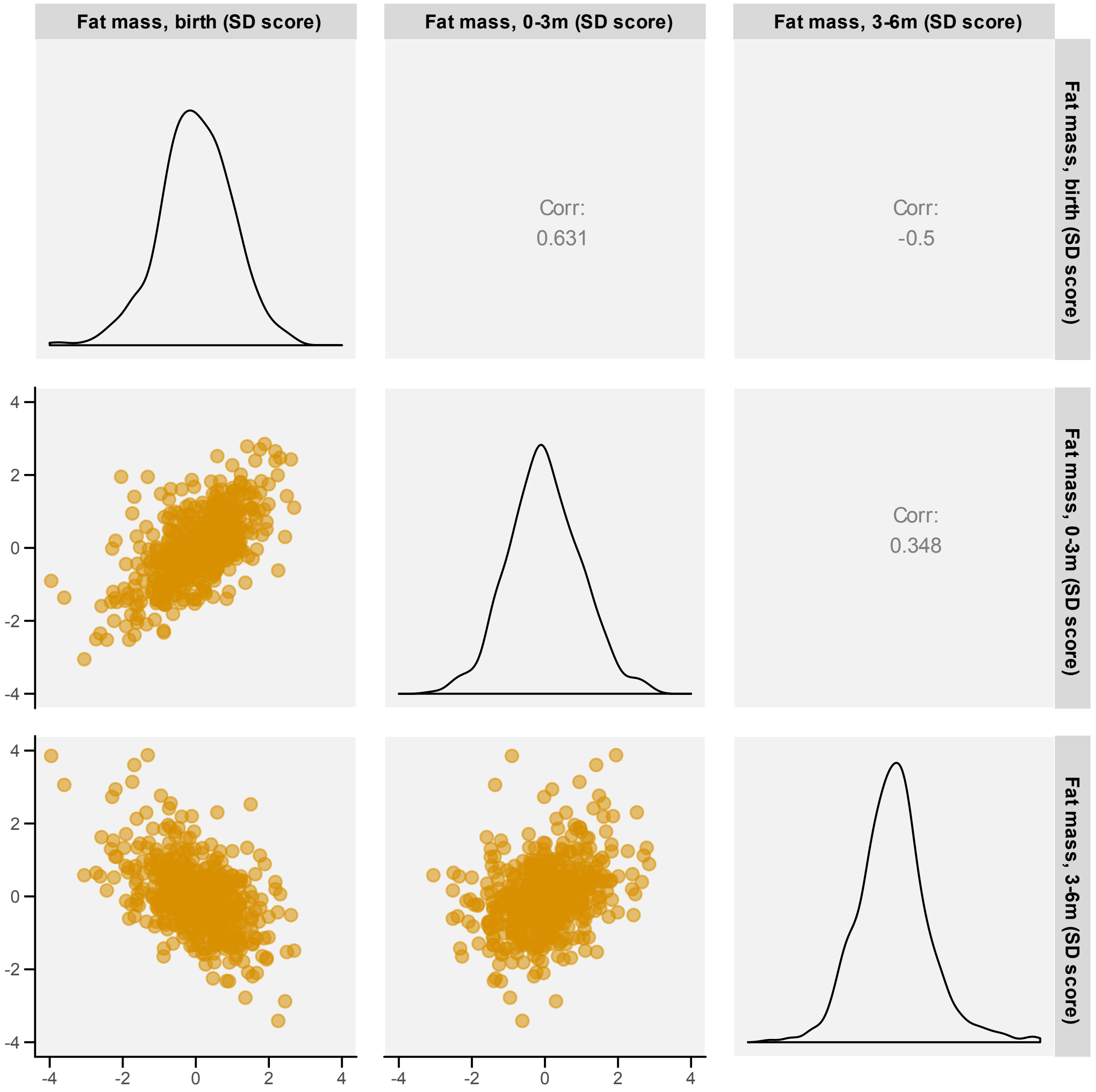

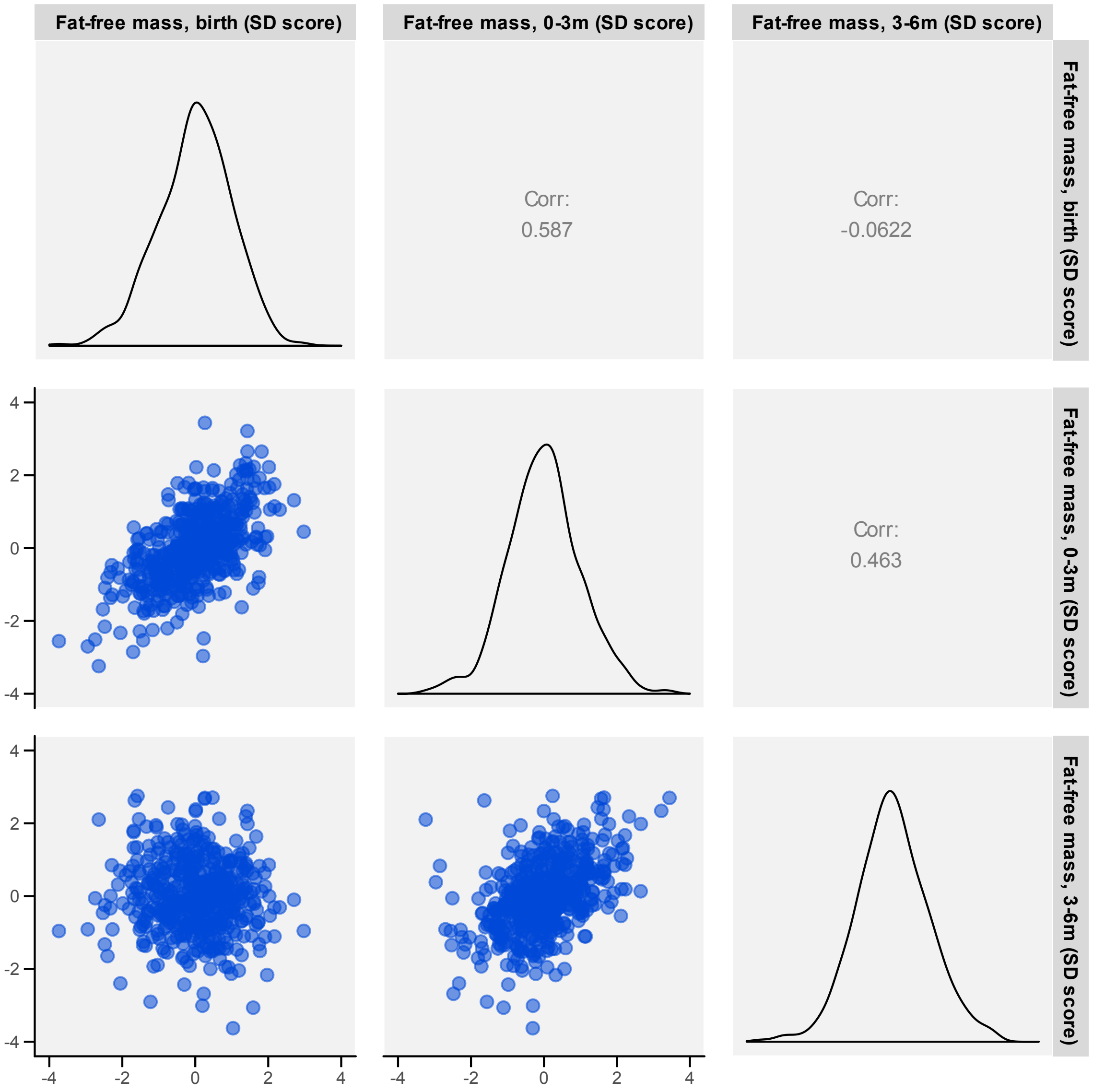

Fat-free mass, birth (SD score)
Fat-free mass, 0-3m (SD score)
Fat-free mass, 3-6m (SD score)
Corr:
0.587
Corr:
-0.0622
Corr:
0.463
Fat-free mass, birth (SD score)
Fat-free mass, 0-3m (SD score)
Fat-free mass, 3-6m (SD score)
4
2
0
-2
-4
-4
-2
0
2
4
