# Supplementary material for: Associations of fat mass and fat-free mass accretion in infancy with body composition and cardiometabolic risk markers at 5 years: The Ethiopian iABC birth cohort study
Source: PLoS Med. 2019 Aug 20;16(8):e1002888. doi: 10.1371/journal.pmed.1002888 (PMC6701744; doi:10.1371/journal.pmed.1002888)
Supplement: S4 Fig — (PDF) [file pmed.1002888.s004.pdf]

S4 Fig. Matrix of model assumption tests of the linear-spline mixed-effect modelling of fat mass and fat-free mass growth velocity. Distribution of outcome variable, residuals plot (residuals regressed on fitted values), histogram of residuals, and Q-Q plot (sample distribution plotted against the theoretical distribution). Model convergence was assessed by checking gradient calculations.

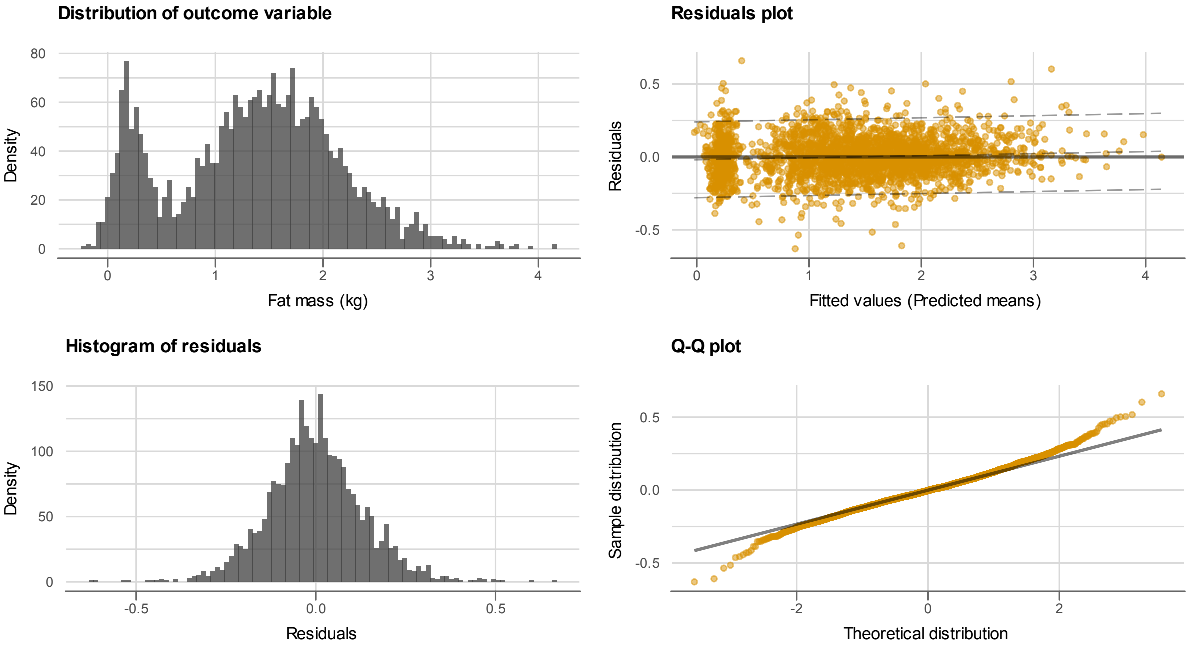

Model specification in R: lmer(formula = Fat mass (kg) ~ Age in months + pmax((Age in months) − 3, 0) + (Age in months + pmax((Age in months) − 3, 0) | Id), REML = FALSE, na.action = na.exclude, data = Data)
Assessing convergence by checking gradient calculations (This number should be <0.001): <0.000001

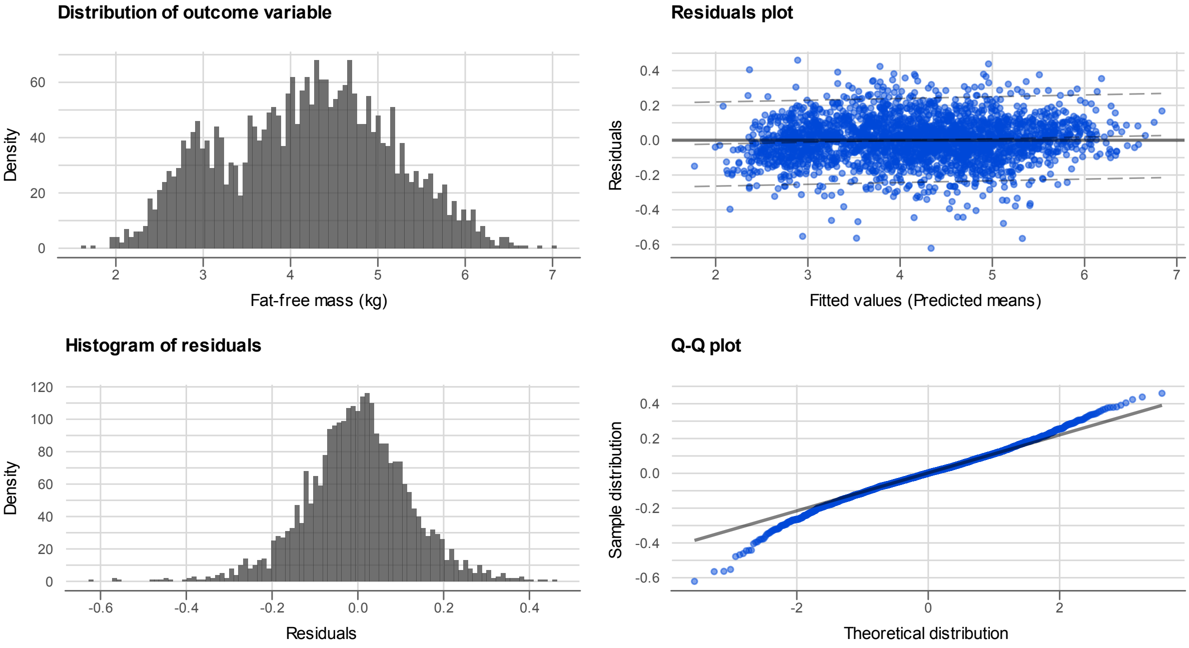

Model specification: : lmer(formula = Fat-free mass (kg) ~ Age in months + pmax((Age in months) − 3, 0) + (Age in months + pmax((Age in months) − 3, 0) | Id), REML = FALSE, na.action = na.exclude, data = Data)
Assessing convergence by checking gradient calculations (This number should be <0.001): <0.000001
