# Supplementary material for: Associations of fat mass and fat-free mass accretion in infancy with body composition and cardiometabolic risk markers at 5 years: The Ethiopian iABC birth cohort study
Source: PLoS Med. 2019 Aug 20;16(8):e1002888. doi: 10.1371/journal.pmed.1002888 (PMC6701744; doi:10.1371/journal.pmed.1002888)
Supplement: S5 Fig — Associations of estimated fat mass and fat-free mass at birth and fat mass and fat-free mass growth velocity in the periods 0–3 and 3–6 months with body composition and cardiometabolic risk markers at 5 years (all analyses adjusted for breastfeeding at 4.5 to 6 months postpartum). (PDF) [file pmed.1002888.s005.pdf]

S5 Fig. Sensitivity analyses of the associations shown in Fig 3. Associations of estimated fat mass and fat-free mass at birth and fat mass and fat-free mass growth velocity in the periods 0-3 and 3-6 months with body composition and cardiometabolic risk markers at 5 years (all analyses adjusted for breastfeeding at 4.5 to 6 months postpartum).

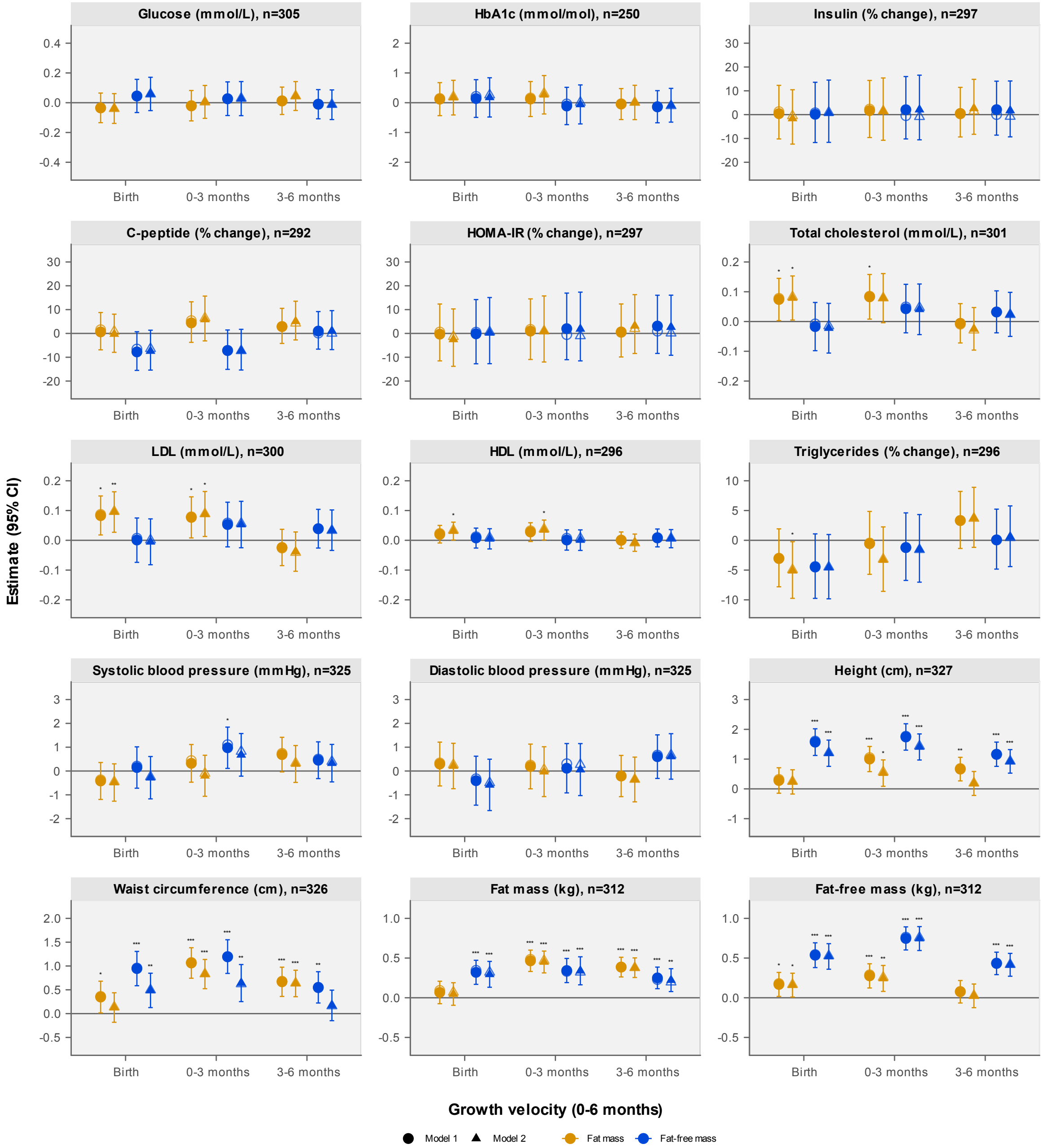

The hollow circles and hollow triangles show the estimates without adjustments for breastfeeding but based on the same subsample of children who have information on breastfeeding. The coefficients (and 95% CIs) displayed in the forest plots were derived from separate multiple linear regression analyses and represent the change in the 5-year outcomes per study population standard deviation increase of estimated fat mass and fat-free mass at birth and fat mass and fat-free mass growth velocity over the periods 0-3 months and 3-6 months. The linear-spline mixed-effects model used to derive the estimated values at birth and growth velocities had one knot point at 3 months, yielding the 2 growth periods 0-3 and 3-6 months. Variables found not to follow a normal distribution (i.e. insulin, C-peptide, HOMA-IR, and triglycerides) were log-transformed prior to the regression analyses. The presented effect estimates for these variables were back-transformed and are shown as percentwise change. Model 1 was adjusted for child's sex, birth order, gestational age at birth, child's exact age at the 5-year visit, maternal age at delivery, maternal postpartum height, maternal educational status, family socioeconomic status (International Wealth Index), and breastfeeding status at 4-6 months postpartum. Model 2 was additionally adjusted for fat mass at the 5-year visit (applies to all outcomes except fat mass and waist circumference, which were adjusted for fat-free mass at the 5-year visit instead of fat mass in model 2). * $P<0.05$, ** $P<0.01$, *** $P<0.001$.
