# Supplementary material for: Associations of fat mass and fat-free mass accretion in infancy with body composition and cardiometabolic risk markers at 5 years: The Ethiopian iABC birth cohort study
Source: PLoS Med. 2019 Aug 20;16(8):e1002888. doi: 10.1371/journal.pmed.1002888 (PMC6701744; doi:10.1371/journal.pmed.1002888)
Supplement: S6 Fig — (PDF) [file pmed.1002888.s006.pdf]

S6 Fig. Sensitivity analyses accounting for multiple testing in model 2 of the associations of estimated fat mass and fat-free mass at birth and fat mass and fat-free mass growth velocity for the periods 0-3 and 3-6 months with body composition and cardiometabolic risk markers at 5 years.

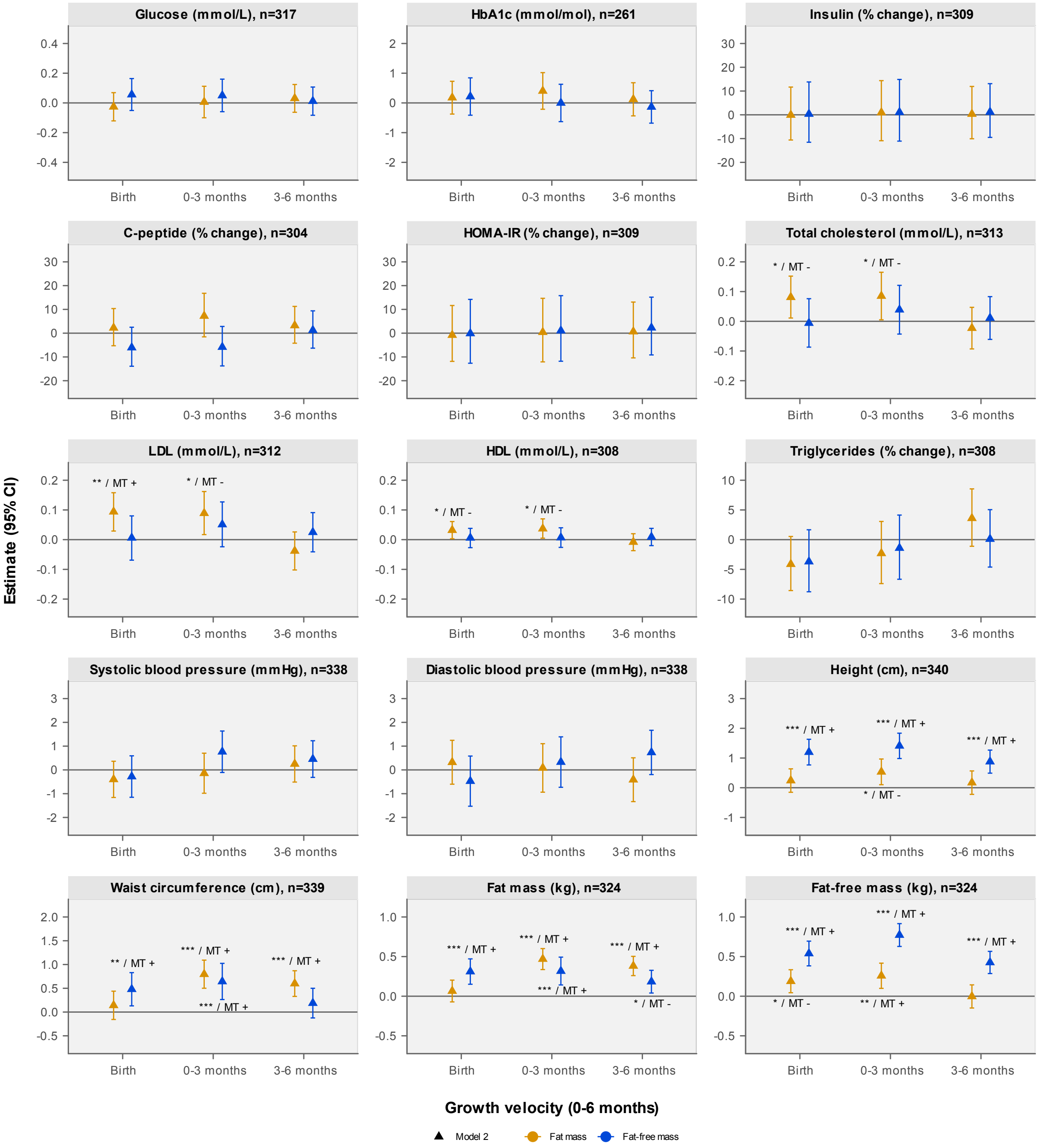

The 5% level of significance was adjusted for multiple testing using the Benjamini–Hochberg method. The significance stars on the left-hand side of the forward slash shows the significance-level before the adjustment and the text on the right-hand side shows if significance remained after adjusting for multiple testing. The designation “MT+” indicates that the significant association remained, and “MT-“ indicates that the association was no longer significant after adjusting for multiple testing. The coefficients (and 95% CIs) displayed in the forest plots were derived from separate multiple linear regression analyses and represent the change in the 5-year outcomes per study population standard deviation increase of estimated fat mass and fat-free mass at birth and fat mass and fat-free mass growth velocity over the periods 0-3 months and 3-6 months. The linear-spline mixed-effects model used to derive the estimated values at birth and growth velocities had one knot point at 3 months, yielding the two growth periods 0-3 and 3-6 months. Variables found not to follow a normal distribution (i.e. insulin, C-peptide, HOMA-IR, and triglycerides) were log-transformed prior to the regression analyses. The presented effect estimates for these variables were back-transformed and are shown as percentwise change. Model 2 was adjusted for child's sex, birth order, gestational age at birth, child’s exact age at the 5-year visit, maternal age at delivery, maternal postpartum height, maternal educational status, family socioeconomic status (International Wealth Index), and fat mass at the 5-year visit (applies to all outcomes except fat mass and waist circumference, which were adjusted for fat-free mass at the 5-year visit instead of fat mass in model 2).
* P<0.05, ** P<0.01, *** P<0.001.
