# Supplementary material for: Associations of fat mass and fat-free mass accretion in infancy with body composition and cardiometabolic risk markers at 5 years: The Ethiopian iABC birth cohort study
Source: PLoS Med. 2019 Aug 20;16(8):e1002888. doi: 10.1371/journal.pmed.1002888 (PMC6701744; doi:10.1371/journal.pmed.1002888)
Supplement: S1 Table — (PDF) [file pmed.1002888.s007.pdf]

**S1 Table** Associations of predicted fat mass and fat-free mass at birth and fat mass and fat-free mass growth velocity in the periods 0-3 and 3-6 months with cardiometabolic markers and body composition at 5 years in the fully adjusted model 2 (exposures in SD units) [1]

| | | Fat mass $_{0\text{-}6\ months}$ | | | | Fat-free mass $_{0\text{-}6\ months}$ | | | |
|---|---|---|---|---|---|---|---|---|---|
| | **n** | **β** | **(95% CI)** | | **p-value** | **β** | **(95% CI)** | | **p-value** |
| **Glucose (mmol/L)** | 305 | | | | | | | | |
| Birth (SD) | | -0.03 | -0.12 | 0.07 | 0.587 | 0.06 | -0.05 | 0.16 | 0.304 |
| 0-3 months (SD) | | 0.01 | -0.10 | 0.11 | 0.915 | 0.05 | -0.06 | 0.16 | 0.365 |
| 3-6 months (SD) | | 0.03 | -0.06 | 0.12 | 0.521 | 0.01 | -0.08 | 0.11 | 0.804 |
| **HbA1c (mmol/mol)** | 250 | | | | | | | | |
| Birth (SD) | | 0.2 | -0.4 | 0.7 | 0.528 | 0.2 | -0.4 | 0.8 | 0.500 |
| 0-3 months (SD) | | 0.4 | -0.2 | 1.0 | 0.199 | -0.0 | -0.6 | 0.6 | 0.998 |
| 3-6 months (SD) | | 0.1 | -0.4 | 0.7 | 0.668 | -0.1 | -0.7 | 0.4 | 0.628 |
| **Insulin (% change)** | 298 | | | | | | | | |
| Birth (SD) | | -0.1 | -10.6 | 11.7 | 0.988 | 0.3 | -11.5 | 13.8 | 0.958 |
| 0-3 months (SD) | | 1.0 | -10.9 | 14.4 | 0.881 | 1.1 | -11.1 | 14.9 | 0.870 |
| 3-6 months (SD) | | 0.3 | -10.1 | 11.9 | 0.954 | 1.2 | -9.5 | 13.1 | 0.838 |
| **C-peptide (% change)** | 293 | | | | | | | | |
| Birth (SD) | | 2.2 | -5.3 | 10.4 | 0.570 | -6.1 | -13.9 | 2.5 | 0.159 |
| 0-3 months (SD) | | 7.2 | -1.5 | 16.8 | 0.109 | -5.8 | -13.8 | 2.8 | 0.180 |
| 3-6 months (SD) | | 3.2 | -4.2 | 11.3 | 0.403 | 1.2 | -6.3 | 9.4 | 0.754 |
| **HOMA-IR (% change)** [2] | 298 | | | | | | | | |
| Birth (SD) | | -0.8 | -11.9 | 11.6 | 0.893 | -0.1 | -12.6 | 14.2 | 0.987 |
| 0-3 months (SD) | | 0.4 | -12.1 | 14.7 | 0.952 | 1.1 | -11.8 | 15.8 | 0.880 |
| 3-6 months (SD) | | 0.7 | -10.4 | 13.1 | 0.911 | 2.3 | -9.2 | 15.2 | 0.708 |
| **Total cholesterol (mmol/L)** | 301 | | | | | | | | |
| Birth (SD) | | 0.08 | 0.01 | 0.15 | 0.025 | -0.01 | -0.09 | 0.08 | 0.889 |
| 0-3 months (SD) | | 0.08 | 0.00 | 0.16 | 0.037 | 0.04 | -0.04 | 0.12 | 0.346 |
| 3-6 months (SD) | | -0.02 | -0.09 | 0.05 | 0.515 | 0.01 | -0.06 | 0.08 | 0.764 |
| **LDL (mmol/L)** | 301 | | | | | | | | |
| Birth (SD) | | 0.09 | 0.03 | 0.16 | 0.005 | 0.01 | -0.07 | 0.08 | 0.878 |
| 0-3 months (SD) | | 0.09 | 0.02 | 0.16 | 0.016 | 0.05 | -0.02 | 0.13 | 0.179 |
| 3-6 months (SD) | | -0.04 | -0.10 | 0.03 | 0.246 | 0.02 | -0.04 | 0.09 | 0.453 |
| **HDL (mmol/L)** | 297 | | | | | | | | |
| Birth (SD) | | 0.03 | 0.00 | 0.06 | 0.028 | 0.01 | -0.03 | 0.04 | 0.738 |
| 0-3 months (SD) | | 0.04 | 0.00 | 0.07 | 0.023 | 0.01 | -0.03 | 0.04 | 0.689 |
| 3-6 months (SD) | | -0.01 | -0.04 | 0.02 | 0.561 | 0.01 | -0.02 | 0.04 | 0.535 |
| **Triglycerides (% change)** | 297 | | | | | | | | |
| Birth (SD) | | -4.1 | -8.6 | 0.5 | 0.082 | -3.7 | -8.8 | 1.7 | 0.172 |
| 0-3 months (SD) | | -2.3 | -7.4 | 3.1 | 0.392 | -1.4 | -6.7 | 4.1 | 0.610 |
| 3-6 months (SD) | | 3.6 | -1.1 | 8.5 | 0.136 | 0.1 | -4.6 | 5.0 | 0.967 |
| **Systolic blood pressure (mmHg)** | 324 | | | | | | | | |
| Birth (SD) | | -0.4 | -1.2 | 0.4 | 0.305 | -0.3 | -1.2 | 0.6 | 0.529 |
| 0-3 months (SD) | | -0.1 | -1.0 | 0.7 | 0.745 | 0.8 | -0.1 | 1.6 | 0.086 |
| 3-6 months (SD) | | 0.3 | -0.5 | 1.0 | 0.517 | 0.5 | -0.3 | 1.2 | 0.245 |

Table continues on the next page.

**S1 Table (continued)** Associations of predicted fat mass and fat-free mass at birth and fat mass and fat-free mass growth velocity in the periods 0-3 and 3-6 months with cardiometabolic markers and body composition at 5 years in the fully adjusted model (exposures in SD units) [1]

| | | Fat mass 0-6 months | | | | Fat-free mass 0-6 months | | | |
|---|---|---|---|---|---|---|---|---|---|
| | n | β | (95% CI) | | p-value | β | (95% CI) | | p-value |
| **Diastolic blood pressure (mmHg)** | 324 | | | | | | | | |
| Birth (SD) | | 0.3 | -0.6 | 1.2 | 0.492 | -0.5 | -1.5 | 0.6 | 0.379 |
| 0-3 months (SD) | | 0.1 | -0.9 | 1.1 | 0.872 | 0.3 | -0.7 | 1.4 | 0.541 |
| 3-6 months (SD) | | -0.4 | -1.3 | 0.5 | 0.379 | 0.7 | -0.2 | 1.7 | 0.121 |
| **Height (cm)** | 324 | | | | | | | | |
| Birth (SD) | | 0.2 | -0.2 | 0.6 | 0.229 | 1.2 | 0.8 | 1.6 | <.001 |
| 0-3 months (SD) | | 0.5 | 0.1 | 1.0 | 0.016 | 1.4 | 1.0 | 1.8 | <.001 |
| 3-6 months (SD) | | 0.2 | -0.2 | 0.6 | 0.393 | 0.9 | 0.5 | 1.3 | <.001 |
| **Waist circumference (cm)** | 324 | | | | | | | | |
| Birth (SD) | | 0.1 | -0.2 | 0.4 | 0.352 | 0.5 | 0.1 | 0.8 | 0.007 |
| 0-3 months (SD) | | 0.8 | 0.5 | 1.1 | <.001 | 0.6 | 0.3 | 1.0 | 0.001 |
| 3-6 months (SD) | | 0.6 | 0.3 | 0.9 | <.001 | 0.2 | -0.1 | 0.5 | 0.236 |
| **Fat mass (kg)** | 324 | | | | | | | | |
| Birth (SD) | | 0.065 | -0.072 | 0.203 | 0.353 | 0.311 | 0.151 | 0.471 | <.001 |
| 0-3 months (SD) | | 0.469 | 0.336 | 0.601 | <.001 | 0.317 | 0.142 | 0.493 | <.001 |
| 3-6 months (SD) | | 0.382 | 0.260 | 0.503 | <.001 | 0.184 | 0.040 | 0.327 | 0.012 |
| **Fat-free mass (kg)** | 324 | | | | | | | | |
| Birth (SD) | | 0.189 | 0.044 | 0.334 | 0.011 | 0.539 | 0.383 | 0.695 | <.001 |
| 0-3 months (SD) | | 0.258 | 0.099 | 0.417 | 0.002 | 0.772 | 0.628 | 0.916 | <.001 |
| 3-6 months (SD) | | -0.003 | -0.149 | 0.143 | 0.971 | 0.426 | 0.286 | 0.566 | <.001 |

[1] The coefficients (and 95% CIs) were derived from separate multiple linear regression analyses and represent the change in the 5-year outcomes per study population standard deviation increase of predicted fat mass and fat-free mass at birth and fat mass and fat-free mass growth velocity in the periods 0-3 months and 3-6 months. Variables found not to follow a normal distribution (i.e. insulin, C-peptide, HOMA-IR, and triglycerides) were log-transformed prior to the regression analyses. The presented effect estimates for these variables were back-transformed and are shown as percentwise change. The presented estimates were adjusted for child's sex, birth order, gestational age at birth, child's exact age at the 5-year visit, maternal age at delivery, maternal postpartum height, maternal educational status, family socioeconomic status (International Wealth Index), and fat mass at the 5-year visit (applies to all outcomes except fat mass and waist circumference, which were adjusted for fat-free mass at the 5-year visit instead of fat mass). [2] Homeostasis model assessment of insulin resistance (HOMA-IR) was calculated as insulin (µU/mL) × glucose (mmol/l) / 22.5. *p < 0.05, **p < 0.01, ***p < 0.001.
