# Supplementary material for: Associations of fat mass and fat-free mass accretion in infancy with body composition and cardiometabolic risk markers at 5 years: The Ethiopian iABC birth cohort study
Source: PLoS Med. 2019 Aug 20;16(8):e1002888. doi: 10.1371/journal.pmed.1002888 (PMC6701744; doi:10.1371/journal.pmed.1002888)
Supplement: S2 Table — (PDF) [file pmed.1002888.s008.pdf]

S2 Table Associations of predicted fat mass and fat-free mass at birth and fat mass and fat-free mass growth velocity in the periods 0-3 and 3-6 months with cardiometabolic markers and body composition at 5 years in the fully adjusted model 2 (exposures in absolute values).[1]

		Fat mass $_{0\text{-}6\ months}$				Fat-free mass $_{0\text{-}6\ months}$			
	**n**	**β**	**(95% CI)**		**p-value**	**β**	**(95% CI)**		**p-value**
**Glucose (mmol/L)**	305								
Birth (100 g)		-0.04	-0.20	0.11	0.587	0.02	-0.02	0.06	0.304
0-3 months (100 g/mo)		0.00	-0.07	0.08	0.915	0.06	-0.08	0.21	0.365
3-6 months (100 g/mo)		0.03	-0.06	0.12	0.521	0.02	-0.12	0.16	0.804
**HbA1c (mmol/mol)**	250								
Birth (100 g)		0.3	-0.6	1.2	0.528	0.1	-0.1	0.3	0.500
0-3 months (100 g/mo)		0.3	-0.2	0.7	0.199	-0.0	-0.8	0.8	0.998
3-6 months (100 g/mo)		0.1	-0.4	0.7	0.668	-0.2	-1.0	0.6	0.628
**Insulin (% change)**	298								
Birth (100 g)		-0.1	-17.0	20.1	0.988	0.1	-4.2	4.6	0.958
0-3 months (100 g/mo)		0.7	-8.0	10.2	0.881	1.4	-14.1	19.7	0.870
3-6 months (100 g/mo)		0.3	-9.7	11.4	0.954	1.7	-13.6	19.7	0.838
**C-peptide (% change)**	293								
Birth (100 g)		3.7	-8.6	17.8	0.570	-2.2	-5.1	0.9	0.159
0-3 months (100 g/mo)		5.2	-1.1	11.9	0.109	-7.5	-17.5	3.7	0.180
3-6 months (100 g/mo)		3.1	-4.1	10.8	0.403	1.8	-9.1	14.0	0.754
**HOMA-IR (% change) [2]**	298								
Birth (100 g)		-1.3	-18.9	20.1	0.893	-0.0	-4.6	4.8	0.987
0-3 months (100 g/mo)		0.3	-8.9	10.4	0.952	1.4	-15.1	21.0	0.880
3-6 months (100 g/mo)		0.6	-10.0	12.6	0.911	3.4	-13.1	22.9	0.708
**Total cholesterol (mmol/L)**	301								
Birth (100 g)		0.14	0.02	0.25	0.025	-0.00	-0.03	0.03	0.889
0-3 months (100 g/mo)		0.06	0.00	0.12	0.037	0.05	-0.06	0.16	0.346
3-6 months (100 g/mo)		-0.02	-0.09	0.04	0.515	0.02	-0.09	0.12	0.764
**LDL (mmol/L)**	301								
Birth (100 g)		0.16	0.05	0.26	0.005	0.00	-0.02	0.03	0.878
0-3 months (100 g/mo)		0.06	0.01	0.12	0.016	0.07	-0.03	0.16	0.179
3-6 months (100 g/mo)		-0.04	-0.10	0.02	0.246	0.04	-0.06	0.13	0.453
**HDL (mmol/L)**	297								
Birth (100 g)		0.05	0.01	0.10	0.028	0.00	-0.01	0.01	0.738
0-3 months (100 g/mo)		0.03	0.00	0.05	0.023	0.01	-0.03	0.05	0.689
3-6 months (100 g/mo)		-0.01	-0.04	0.02	0.561	0.01	-0.03	0.06	0.535
**Triglycerides (% change)**	297								
Birth (100 g)		-6.8	-13.8	0.9	0.082	-1.3	-3.2	0.6	0.172
0-3 months (100 g/mo)		-1.7	-5.4	2.2	0.392	-1.8	-8.6	5.4	0.610
3-6 months (100 g/mo)		3.5	-1.1	8.2	0.136	0.1	-6.7	7.5	0.967
**Systolic blood pressure (mmHg)**	324								
Birth (100 g)		-0.7	-1.9	0.6	0.305	-0.1	-0.4	0.2	0.529
0-3 months (100 g/mo)		-0.1	-0.7	0.5	0.745	1.0	-0.1	2.1	0.086
3-6 months (100 g/mo)		0.2	-0.5	1.0	0.517	0.7	-0.5	1.8	0.245

Table continues on the next page.

S2 Table (continued) Associations of predicted fat mass and fat-free mass at birth and fat mass and fat-free mass growth velocity in the periods 0-3 and 3-6 months with cardiometabolic markers and body composition at 5 years in the fully adjusted model 2 (exposures in absolute values).[1]

		Fat mass $_{0\text{-}6\ months}$				Fat-free mass $_{0\text{-}6\ months}$			
	**n**	**Β**	**(95% CI)**		**p-value**	**β**	**(95% CI)**		**p-value**
**Diastolic blood pressure (mmHg)**	324								
Birth (100 g)		0.5	-1.0	2.1	0.492	-0.2	-0.5	0.2	0.379
0-3 months (100 g/mo)		0.1	-0.7	0.8	0.872	0.4	-0.9	1.8	0.541
3-6 months (100 g/mo)		-0.4	-1.3	0.5	0.379	1.1	-0.3	2.4	0.121
**Height (cm)**	324								
Birth (100 g)		0.4	-0.3	1.1	0.229	0.4	0.3	0.6	<.001
0-3 months (100 g/mo)		0.4	0.1	0.7	0.016	1.8	1.3	2.4	<.001
3-6 months (100 g/mo)		0.2	-0.2	0.5	0.393	1.3	0.7	1.9	<.001
**Waist circumference (cm)**	324								
Birth (100 g)		0.2	-0.3	0.7	0.352	0.2	0.0	0.3	0.007
0-3 months (100 g/mo)		0.6	0.4	0.8	<.001	0.8	0.3	1.3	0.001
3-6 months (100 g/mo)		0.6	0.3	0.8	<.001	0.3	-0.2	0.7	0.236
**Fat mass (kg)**	324								
Birth (100 g)		0.108	-0.120	0.337	0.353	0.109	0.053	0.165	<.001
0-3 months (100 g/mo)		0.339	0.243	0.435	<.001	0.412	0.184	0.640	<.001
3-6 months (100 g/mo)		0.367	0.250	0.484	<.001	0.269	0.059	0.478	0.012
**Fat-free mass (kg)**	324								
Birth (100 g)		0.314	0.074	0.554	0.011	0.189	0.134	0.243	<.001
0-3 months (100 g/mo)		0.187	0.072	0.302	0.002	1.002	0.815	1.189	<.001
3-6 months (100 g/mo)		-0.003	-0.143	0.138	0.971	0.624	0.419	0.829	<.001

[1] The coefficients (and 95% CIs) were derived from separate multiple linear regression analyses and represent the change in the 5-year outcomes per 100 g increase in predicted fat mass and fat-free mass at birth and 100 g/months increase fat mass and fat-free mass in the periods 0-3 months and 3-6 months. Variables found not to follow a normal distribution (i.e. insulin, C-peptide, HOMA-IR, and triglycerides) were log-transformed prior to the regression analyses. The presented effect estimates for these variables were back-transformed and are shown as percentwise change. The presented estimates were adjusted for child's sex, birth order, gestational age at birth, child's exact age at the 5-year visit, maternal age at delivery, maternal postpartum height, maternal educational status, family socioeconomic status (International Wealth Index), and fat mass at the 5-year visit (applies to all outcomes except fat mass and waist circumference, which were adjusted for fat-free mass at the 5-year visit instead of fat mass). [2] Homeostasis model assessment of insulin resistance (HOMA-IR) was calculated as insulin (µU/mL) × glucose (mmol/l) / 22.5. *p < 0.05, **p < 0.01, ***p < 0.001.
