# Supplementary material for: Associations of fat mass and fat-free mass accretion in infancy with body composition and cardiometabolic risk markers at 5 years: The Ethiopian iABC birth cohort study
Source: PLoS Med. 2019 Aug 20;16(8):e1002888. doi: 10.1371/journal.pmed.1002888 (PMC6701744; doi:10.1371/journal.pmed.1002888)
Supplement: S3 Table — (PDF) [file pmed.1002888.s009.pdf]

S3 Table Comparison of background characteristics of the mother-child pairs attending the 5-year follow-up visit with those not attending [1]

	**Full sample** (n = 632)	**Not attending the visit at 5 years** (n = 292)	**Attending the visit at 5 years** (n = 340)	***p*-value** [2]	**Missing, n**
**Maternal characteristics**					
Age at birth (years)	24.1 (4.6)	23.7 (4.5)	24.5 (4.7)	0.022	11
Postpartum height (cm)	157.5 (6.0)	158.1 (5.7)	157.1 (6.1)	0.027	37
Postpartum body mass index (kg/m^2)	22.28 (3.43)	22.43 (3.27)	22.20 (3.52)	0.465	105
Birth order of current child (%)					
First	55.2	62.1	49.6		
Second	23.9	20.6	26.7		
Third or above	20.9	17.4	23.8	0.007	9
Breastfeeding status at 4 to 6 months post-partum (%)					
Exclusive	14.3	17.9	12.5		
Almost exclusive (water given)	22.7	25.6	21.3		
Predominant	57.6	51.9	60.4		
Partial or no	5.4	4.5	5.8	0.195	148
Maternal education (%)					
No school	7.0	7.0	7.0		
Some primary school	45.5	46.0	45.2		
Completed primary school	15.2	14.4	15.8		
Completed secondary school	18.4	17.5	19.1		
Higher education	13.9	15.1	12.9	0.915	6
Socioeconomic status (International Wealth Index)	45.7 (18.2)	46.0 (19.6)	45.5 (17.0)	0.779	13
**Child characteristics at birth**					
Gender (female)	50.6	51.2	50.1	0.853	0
Gestational age (weeks)	39.0 (1.0)	39.0 (0.9)	39.0 (1.0)	0.864	0
Weight (kg)	3.03 (0.42)	3.01 (0.43)	3.05 (0.40)	0.338	0
Length (cm)	49.1 (2.0)	49.0 (2.1)	49.2 (1.9)	0.424	0
Fat mass (kg)	0.22 (0.16)	0.22 (0.15)	0.22 (0.16)	0.792	3
Fat-free mass (kg)	2.82 (0.33)	2.81 (0.35)	2.83 (0.32)	0.475	3
Low birth weight (%) [3]	10.0	11.0	9.1	0.507	0

[1] Data are mean (SD) for continuous normally distributed variables and percentages for categorical variables. [2] Differences between groups were calculated by One-way ANOVA F-test for continuous variables, Pearson's Chi-Square test of independence for categorical variables with expected counts ≥5 in all cells and Fisher's exact test of independence for categorical variables with expected counts in any cell <5. [3] Low birth weight is defined as birth weight <2500 g.
