# Supplementary material for: Associations of fat mass and fat-free mass accretion in infancy with body composition and cardiometabolic risk markers at 5 years: The Ethiopian iABC birth cohort study
Source: PLoS Med. 2019 Aug 20;16(8):e1002888. doi: 10.1371/journal.pmed.1002888 (PMC6701744; doi:10.1371/journal.pmed.1002888)
Supplement: S1 Text — (PDF) [file pmed.1002888.s010.pdf]

Associations of fat mass and fat-free mass accretion in infancy with body composition and cardiometabolic risk markers at 5 years: The Ethiopian iABC birth cohort study

STROBE Statement—checklist of items that should be included in reports of observational studies

	Item No	Recommendation	Section	Paragraph/sub-section
**Title and abstract**	1	(*a*) Indicate the study's design with a commonly used term in the title or the abstract	Title page<br>Abstract	NA
		(*b*) Provide in the abstract an informative and balanced summary of what was done and what was found	Abstract	Methods and Findings
**Introduction**				
Background/rationale	2	Explain the scientific background and rationale for the investigation being reported	Introduction	First, second and third paragraph
Objectives	3	State specific objectives, including any prespecified hypotheses	Introduction	Third paragraph
**Methods**				
Study design	4	Present key elements of study design early in the paper	Methods	Study setting and participants
Setting	5	Describe the setting, locations, and relevant dates, including periods of recruitment, exposure, follow-up, and data collection	Methods	Study setting and participants
Participants	6	(*a*) *Cohort study*—Give the eligibility criteria, and the sources and methods of selection of participants. Describe methods of follow-up<br>*Case-control study*—Give the eligibility criteria, and the sources and methods of case ascertainment and control selection. Give the rationale for the choice of cases and controls<br>*Cross-sectional study*—Give the eligibility criteria, and the sources and methods of selection of participants	Methods	Study setting and participants

		(*b*) *Cohort study*—For matched studies, give matching criteria and number of exposed and unexposed<br>*Case-control study*—For matched studies, give matching criteria and the number of controls per case	NA	NA
Variables	7	Clearly define all outcomes, exposures, predictors, potential confounders, and effect modifiers. Give diagnostic criteria, if applicable	Methods	Data collection:<br>Anthropometry and body composition in infancy and early childhood; Blood pressure at 5 years; Cardiometabolic markers at 5 years; Covariates
Data sources/ measurement	8*	For each variable of interest, give sources of data and details of methods of assessment (measurement). Describe comparability of assessment methods if there is more than one group	Methods	Data collection:<br>Anthropometry and body composition in infancy and early childhood; Blood pressure at 5 years; Cardiometabolic markers at 5 years; Covariates
Bias	9	Describe any efforts to address potential sources of bias	Methods	Statistical methods:<br>Fat- and fat-free mass accretion in early life; Associations of fat- and fat-free mass accretion in early life with body composition and cardiometabolic markers at 5 years
Study size	10	Explain how the study size was arrived at	Results	First paragraph; Fig 1
Quantitative variables	11	Explain how quantitative variables were handled in the analyses. If applicable, describe which groupings were chosen and why	Methods	Data collection:<br>Anthropometry and body composition in infancy and early childhood; Blood pressure at 5 years; Cardiometabolic markers at 5 years; Covariates;<br><br>Statistical methods:<br>Fat- and fat-free mass accretion in early life

Statistical methods	12	(*a*) Describe all statistical methods, including those used to control for confounding	Methods	Statistical methods:<br>Fat- and fat-free mass accretion in early life;<br>Associations of fat- and fat-free mass accretion in early life with body composition and cardiometabolic markers at 5 years
		(*b*) Describe any methods used to examine subgroups and interactions	Methods	Statistical methods:<br>Associations of fat- and fat-free mass accretion in early life with body composition and cardiometabolic markers at 5 years
		(*c*) Explain how missing data were addressed	Methods<br><br>Supporting Information	Statistical methods:<br>Associations of fat- and fat-free mass accretion in early life with body composition and cardiometabolic markers at 5 years<br><br>S3 Text
		(*d*) *Cohort study*—If applicable, explain how loss to follow-up was addressed<br>*Case-control study*—If applicable, explain how matching of cases and controls was addressed<br>*Cross-sectional study*—If applicable, describe analytical methods taking account of sampling strategy	Discussion<br><br>Supporting Information	Strengths and limitations:<br>Second paragraph<br><br>S3 Table
		(*e*) Describe any sensitivity analyses	Methods<br><br>Supporting Information	Statistical methods:<br>Associations of fat- and fat-free mass accretion in early life with body composition and cardiometabolic markers at 5 years<br><br>S5 Fig.; S6 Fig.

Continued on next page

	Item No	Recommendation	Section	Paragraph/sub-section
**Results**				
Participants	13*	(a) Report numbers of individuals at each stage of study—eg numbers potentially eligible, examined for eligibility, confirmed eligible, included in the study, completing follow-up, and analysed	Methods<br>Results	Study setting and participants<br>First paragraph; Fig 1
		(b) Give reasons for non-participation at each stage	Results	First paragraph; Fig 1
		(c) Consider use of a flow diagram	Results	Fig 1
Descriptive data	14*	(a) Give characteristics of study participants (eg demographic, clinical, social) and information on exposures and potential confounders	Results	Second paragraph; Table 1
		(b) Indicate number of participants with missing data for each variable of interest	Results	Table 1
		(c) *Cohort study*—Summarise follow-up time (eg, average and total amount)	Results	In Table 1 on the line "Age at 5-year visit (months)"
Outcome data	15*	*Cohort study*—Report numbers of outcome events or summary measures over time	Results	Table 2
		Case-control study—Report numbers in each exposure category, or summary measures of exposure	NA	NA
		Cross-sectional study—Report numbers of outcome events or summary measures	NA	NA
Main results	16	(*a*) Give unadjusted estimates and, if applicable, confounder-adjusted estimates and their precision (eg, 95% confidence interval). Make clear which confounders were adjusted for and why they were included	Results	Figure 3; S1 Table; S2 Table
		(*b*) Report category boundaries when continuous variables were categorized	NA	NA
		(*c*) If relevant, consider translating estimates of relative risk into absolute risk for a meaningful time period	NA	NA

Other analyses	17	Report other analyses done—eg analyses of subgroups and interactions, and sensitivity analyses	Supporting Information	S5 Fig.; S6 Fig.
**Discussion**				
Key results	18	Summarise key results with reference to study objectives	Discussion	First paragraph
Limitations	19	Discuss limitations of the study, taking into account sources of potential bias or imprecision. Discuss both direction and magnitude of any potential bias	Discussion	Strengths and limitations: Second paragraph
Interpretation	20	Give a cautious overall interpretation of results considering objectives, limitations, multiplicity of analyses, results from similar studies, and other relevant evidence	Discussion	Conclusion: Last paragraph
Generalisability	21	Discuss the generalisability (external validity) of the study results	Discussion	Strengths and limitations: Second paragraph
**Other information**				
Funding	22	Give the source of funding and the role of the funders for the present study and, if applicable, for the original study on which the present article is based	Stated in the article submission form	NA

*Give information separately for cases and controls in case-control studies and, if applicable, for exposed and unexposed groups in cohort and cross-sectional studies.

Note: An Explanation and Elaboration article discusses each checklist item and gives methodological background and published examples of transparent reporting. The STROBE checklist is best used in conjunction with this article (freely available on the Web sites of PLoS Medicine at http://www.plosmedicine.org/, Annals of Internal Medicine at http://www.annals.org/, and Epidemiology at http://www.epidem.com/). Information on the STROBE Initiative is available at www.strobe-statement.org.
