# Supplementary material for: Associations of fat mass and fat-free mass accretion in infancy with body composition and cardiometabolic risk markers at 5 years: The Ethiopian iABC birth cohort study
Source: PLoS Med. 2019 Aug 20;16(8):e1002888. doi: 10.1371/journal.pmed.1002888 (PMC6701744; doi:10.1371/journal.pmed.1002888)
Supplement: S2 Text — (PDF) [file pmed.1002888.s011.pdf]

BODY COMPOSITION TRAJECTORIES AND METABOLIC RISK IN AFRICAN CHILDREN

Abstract

Growth patterns in early life are important predictors of later risk of Type 2 Diabetes (T2D), cardiovascular disease (CVD) and obesity, particular in low and middle-income countries facing a rapid epidemiological and nutritional transition through adaptations to Western lifestyles. Using advanced body composition data from a unique birth cohort of Ethiopian infants, the study aims to identify patterns in fat- and lean mass growth in early life that predispose changes in risk markers of T2D and CVD at 5 years. This is crucial for early identification of T2D and CVD risk groups, in order to initiate timely prevention.

Collaborating partners

University of Copenhagen *Steno Diabetes Center* *Jimma University*

Background

Type 2 Diabetes (T2D) and cardiovascular disease (CVD) are among the leading causes of death and disability worldwide (1), and more than 80% of deaths related to these and other non-communicable diseases (NCDs) are occurring in low- and middle-income countries (LMIC) (2). Moreover, the prevalence of childhood overweight and obesity is rapidly increasing in many LMIC, which is expected to further accelerate the on-going T2D and CVD epidemic in these countries (3). The heavy burden of NCDs coincides with a high incidence of fetal- and child undernutrition, which continues to impair health and development for millions of children (4). In addition to the short-term consequences, such as increased mortality from infectious diseases (5), undernutrition in early life has been found to substantially increase the risk of T2D, CVD and obesity later in life (6-10). This relation is commonly known as developmental origins of health and disease (DOHaD), where environmental cues in fetal life and early infancy, such as nutritional deprivation, cause lasting metabolic alterations during early growth. While these metabolic adaptations improve the immediate survival by preserving energy supply to the growing brain (11, 12), alterations may also promote excess weight gain and increase the susceptibility to metabolic diseases later in life. This is particularly the case when metabolic adaptation to the environmental cues in early life are mismatched with nutritional excess through the lifecourse (13-16) and a number of studies have found that such detrimental metabolic adaptations are manifesting themselves already in early childhood (17-20).

Most research on the relation between early growth, nutritional status and later risk of disease use simple anthropometric indices such as weight, length and skinfolds as indicators of nutritional status, fetal- and infant growth (20-22). However, these indicators do not reveal whether health consequences of suboptimal growth in infancy and early childhood are ascribed to deficits in fat- or lean mass. Infant and childhood deficits in fat- or lean mass are likely to have completely different implications for later health status due to their different metabolic properties (23, 24), and it has been suggested that the distinct properties of fat- and lean mass rather than simple anthropometric indices, play a key role in the link between early growth and subsequent risk of disease (24). In a cohort of children from Spain, assessed with DXA-scans, small for gestational age children had higher abdominal fat mass accretion, between 2 and 4 years, and higher fasting insulin at 4 years, compared to average for gestational age children, despite having the same weight, height and body mass index at 2, 3, and 4 years (19). A study from India found that low birth weight was associated with reduced levels of lean mass and increased levels of fat mass, and hypothesized that the reduced muscle mass could promote insulin resistance (25). Bavdekar et al. (17) studied insulin resistance syndrome in 8 year old Indian children and found that the highest levels of insulin resistance and LDL cholesterol was found in children who were born with low birth weight but had the highest fat mass at 8 years.

These findings are early indications that fat- and lean- mass growth could have very different effects on risk markers of T2D and CVD even in childhood. However, they are based on small numbers and crude assessment of body size. Very few studies have investigated how infant and childhood differences in fat- and lean mass growth translate into T2D and CVD risk using appropriate measurements of fat- and lean mass growth. There is a complete lack of data from Africa and other LMIC settings, although these populations have amongst the highest fertility rates worldwide and their future health is likely to be particularly sensitive to DOHaD effects from an on-going nutritional transition (26).

We therefore aim to test the hypothesis that distinct infant fat- and lean mass growth patterns are associated with childhood markers of insulin resistance, glucose intolerance and CVD risk in a LMIC setting.

Based on an unique African Infant Anthropometry and Body Composition cohort (The iABC Ethiopia cohort) of more than 400 infants with accurate repeated measurements of body composition from birth through infancy, we have already shown a large variation in fat- and lean mass at birth to similar birth weights (27), and identified very distinct growth patterns in fat- and lean mass through infancy (28). With this proposal, we suggest analysing 5-year follow-up data from this cohort with the following objectives.

Objective

We aim to study the relationship between body composition growth in infancy (0-6 months of age), and markers of insulin resistance, glucose intolerance and cardiovascular risk in 5-year-old Ethiopian children.

Methods

Research setting

Data for the present cohort study is currently being collected at Jimma University Specialized Hospital (JUSH) in Jimma, Ethiopia, through the long-standing research collaboration of Jimma University and University of Copenhagen Alliance on Nutrition (JUCAN). The JUCAN collaboration uses gold standard methods to collect data on maternal and child growth, and markers of metabolic diseases. To assess infant, child and maternal body composition accurately, we run one of few combined Peapod and Bodpod systems (COSMED, Rome, Italy) in Africa and have extensive experience in stable isotope assessment of body composition in healthy and sick children and adults, through collaboration with the International Atomic Energy Agency.

Study design

The proposed study will analyse the 5-year follow-up of the iABC cohort that at baseline comprised 618 infants born at JUSH between January 2009 and June 2012 (table 1). All mothers who were residents in Jimma, and who gave birth to a child with a birth weight of ≥1500 gram were offered to have their child included in the first study phase entitled iABC-1. Of those eligible, almost 90% consented to participate. The infants included in iABC-1 were examined within 24 hours after delivery, and again after 1.5, 2.5, 3.5, 4.5 and 6 months of age, using the Peapod system, an infant air-displacement plethysmograph. A detailed description of the data collected in iABC-1 can be found elsewhere (27-29). To further strengthen the baseline assessment, body composition assessment using the Peapod was validated in a subsample using the isotope dilution technique (28). The combination of these state-of-the-art methods allows for very accurate and precise assessment of fat- and lean mass in infants <8 kg, and to the authors knowledge, this is the only setup of its kind in a low-income country setting.

The present study aims to examine the relationship between body composition in early life and markers of insulin resistance, glucose intolerance and CVD risk in 5-year-old Ethiopian children. Follow-up at 3, 4 and 5 years of age is on-going, and we are expecting to recruit a total of 400 children at 5-years.

TABLE 1
Cohort overview

**Study**	**iABC-1** Jan 2009 - Jun 2012						**iABC-3** Jan 2012-Dec 2016		
Age in months	Birth	1.5	2.5	3.5	4.5	6	36	48	60
Visit number	1	2	3	4	5	6	10	11	12
Number examined	618	411	426	420	389	378	226*	330*	224*

* Follow-up is on going. We are expecting to recruit a total of 350-400 children at 60 months.

Data collection at the 5-year examination

Anthropometric measurements: Weight will be measured to the nearest 0.01 kg using a Tanita BD-815U pediatric scale or a Tanita BWB-800S depending on age and size (Tanita Corp., Arlington Heights, USA). Height will be measured to the nearest 0.1 cm using a Seca 213 portable height measurer (SECA, Hamburg, Germany). Head-, abdominal- and mid-upper-arm-circumference (MUAC) will be measured to the nearest 0.1 cm, and triceps- and subscapular skinfold thickness to the nearest 0.2 mm using a non-stretchable measuring tape and Harpenden calliper (CMS instruments, London, UK), respectively.

Body composition assessment: Body composition at 5-years is expected to mediate the relationship between body composition growth in infancy and childhood markers of insulin resistance, glucose intolerance and CVD risk. Thus, body composition at 5-years will be determined using the Bodpod system - an air-displacement plethysmograph (COSMED, Rome, Italy). The Bodpod system is a relatively novel method for very accurate, fast and safe assessment of body composition in children and adults (30-32). In brief, the Bodpod system measures weight and volume of the child to calculate total body density. Since fat and lean mass differs in density, assessment of total body density may be used to contribute body weight to either fat- or lean mass, assuming known values of fat- and lean mass density. While the density of fat mass is relatively constant, the density of lean mass may vary across gender, age- and population groups, mainly due to differences in lean mass hydration. Thus, we will assess total body water in a subsample using the isotope dilution technique, allowing a division of the body in 3 compartments; fat mass, water and dry lean tissue. The combination of these methods allows very accurate measurements of fat- and lean mass.

Blood sampling and markers of insulin resistance, glucose intolerance and CVD risk (lipids and blood pressure): A 2 ml venous blood sample will be collected. Conventional blood slides will be made and examined for malaria parasites. Blood haemoglobin and fasting blood glucose (FBG) will be determined using HemoCue and HemoCue Glucose System (HemoCue, Ängelholm, Sweden), respectively. Glycosylated haemoglobin (HbA1c will be measured on whole blood samples using the fast ion-exchange resin separation method (HUMAN, Wiesbaden, Germany). Serum will be collected, aliquoted in 3x0.4 ml and frozen at -80 degress celcius until analysed. Total cholesterol (TC), and triglyceride concentrations will be measured with enzymatic colorimetric technique (HUMAN, Wiesbaden, Germany). HDL cholesterol will be isolated by precipitation with sodium phosphotungstate and magnesium chloride solution (33). LDL cholesterol will be calculated according to Friedewald's formula (34), and serum insulin concentration will be assessed using a radioimmunoassay kit. Based on the glucose and insulin measurements, the homeostasis model assessment of insulin resistance index (HOMA-IR) will be estimated as a measure of insulin sensitivity, and a metabolic score will be calculated as described by Corvalán et. al. (20). Blood pressure will be measured in duplicates using digital Spot Vital Signs Lxi (Welch Allyn Inc., Skaneateles Falls, USA).

Ethical considerations

Ethical permission for the present 5-year follow-up study has been granted from Jimma University and the National Health Research Ethics Review Committee in Ethiopia. Prior to obtaining written informed consent, written and oral information in the local languages are given to the parents or caretakers of all eligible participants. There are no risks associated with the examinations and only 2 ml of venous blood will be taken from the child. Any serious medical conditions observed by the research nurses during examination of the children will be addressed according to local guidelines.

Significance of the research project

The majority of the current DOHaD research relies on simple anthropometric indices like weight, height and skinfolds to describe the growth trajectories in childhood. There are very few research groups working in LMICs that have the setup and expertise to apply advanced body composition techniques to assess growth in infancy and childhood. The data being collected for this research project is several years ahead of the work of other research groups working in this area in LMICs, and the quality of the cohort is proven through publications in leading journals within the research field (28, 29). Encouraged by emerging evidence from European and Asian cohorts, we aim to comprehensively describe growth in early life and its relationship with markers of insulin resistance, glucose intolerance and CVD risk in childhood. An examination of childhood markers of T2D and CVD risk in a population with unique objective data on body composition will yield completely new insights into the role of fat and lean mass in programming of T2D, CVD and later obesity. Knowledge on the link between fetal and infant fat and lean growth and childhood markers of T2D and CVD risk may translate into improved prevention and treatment of inadequate or accelerated childhood growth. Identification of early-life indicators that can accurately identify individuals at increased risk of T2D, CVD and obesity later in life has game changing potential in LMIC countries where the future treatment of T2D and CVD is a serious threat to health care systems. Information like this may allow health care systems in developed and developing countries to take early preventive actions against these diseases later in life. Further, there is a potential to change current practice by highlighting that future treatment of inadequate childhood growth may not only consider the short-term consequences but also consider that interventions to alleviate inadequate growth in childhood may affect later risk of T2D and CVD. This is particularly important in settings where both growth faltering and excessive growth is common and concomitant. Currently undergoing a rapid epidemiological and nutritional transition, through adaptations to Western lifestyles, and thus particularly sensitive to the adverse effects of early-life programming, Ethiopia provides the ideal research setting to study the antecedents of adult diseases like T2D and CVD.

References

1. Murray CJL, Vos T, Lozano R, Naghavi M, Flaxman AD, Michaud C, Ezzati M, Shibuya K, Salomon JA, Abdalla S, et al. Disability-adjusted life years (DALYs) for 291 diseases and injuries in 21 regions, 1990–2010: a systematic analysis for the Global Burden of Disease Study 2010. Lancet 2012;380:2197-223.
2. Abegunde DO, Mathers CD, Adam T, Ortegon M, Strong K. The burden and costs of chronic diseases in low-income and middle-income countries. Lancet 2007;370:1929-38.
3. Ng M, Fleming T, Robinson M, Thomson B, Graetz N, Margono C, Mullany EC, Biryukov S, Abbafati C, Abera SF, et al. Global, regional, and national prevalence of overweight and obesity in children and adults during 1980-2013: a systematic analysis for the Global Burden of Disease Study 2013. Lancet 2014;384:766-81.
4. Black RE, Victora CG, Walker SP, Bhutta ZA, Christian P, de Onis M, Ezzati M, Grantham- McGregor S, Katz J, Martorell R, et al. Maternal and child undernutrition and overweight in low-income and middle-income countries. Lancet 2013;382:427-51.
5. Olofin I, McDonald CM, Ezzati M, Flaxman S, Black RE, Fawzi WW, Caulfield LE, Danaei G, pooling) NIMSac. Associations of suboptimal growth with all-cause and cause-specific mortality in children under five years: a pooled analysis of ten prospective studies. PLoS One 2013;8:e64636.
6. Barker DJP, Eriksson JG, Forsén T, Osmond C. Fetal origins of adult disease: strength of effects and biological basis. Int J Epidemiol 2002;31:1235-9.
7. Victora CG, Adair L, Fall C, Hallal PC, Martorell R, Richter L, Sachdev HS. Maternal and child undernutrition: consequences for adult health and human capital. Lancet 2008;371:340-57.
8. Eriksson JG, Forsén T, Tuomilehto J, Osmond C, Barker DJ. Early growth and coronary heart disease in later life: longitudinal study. BMJ (Clinical research ed) 2001;322:949-53.
9. Hales CN, Barker DJ. Type 2 (non-insulin-dependent) diabetes mellitus: the thrifty phenotype hypothesis. Diabetologia 1992;35:595-601.
10. Barker D. Mothers, Babies and Health in Later Life. London: Churchill Livingstone, 1998.
11. Leonard WR, Robertson ML, Snodgrass JJ, Kuzawa CW. Metabolic correlates of hominid brain evolution. Comparative Biochemistry and Physiology Part A: Molecular & Integrative Physiology 2003;136:5-15.
12. Kuzawa CW, Chugani HT, Grossman LI, Lipovich L, Muzik O, Hof PR, Wildman DE, Sherwood CC, Leonard WR, Lange N. Metabolic costs and evolutionary implications of human brain development. Proc Natl Acad Sci U S A 2014;111:13010-5.
13. Wells JCK. The thrifty phenotype: An adaptation in growth or metabolism? Am J Hum Biol 2011;23:65-75.
14. Gluckman PD, Hanson MA, Beedle AS. Early life events and their consequences for later disease: a life history and evolutionary perspective. American journal of human biology: the official journal of the Human Biology Council 2007;19:1-19.
15. Bateson P, Barker D, Clutton-Brock T, Deb D, D'Udine B, Foley RA, Gluckman P, Godfrey K, Kirkwood T, Lahr MM, et al. Developmental plasticity and human health. Nature 2004;430:419-21.

16. Metcalfe NB, Monaghan P. Compensation for a bad start: grow now, pay later? Trends Ecol Evol 2001;16:254-60.

17. Bavdekar A, Yajnik CS, Fall CH, Bapat S, Pandit AN, Deshpande V, Bhave S, Kellingray SD, Joglekar C. Insulin resistance syndrome in 8-year-old Indian children: small at birth, big at 8 years, or both? Diabetes 1999;48:2422-9.

18. Joglekar CV, Fall CHD, Deshpande VU, Joshi N, Bhalerao A, Solat V, Deokar TM, Chougule SD, Leary SD, Osmond C, et al. Newborn size, infant and childhood growth, and body composition and cardiovascular disease risk factors at the age of 6 years: the Pune Maternal Nutrition Study. International journal of obesity and related metabolic disorders: journal of the International Association for the Study of Obesity 2007;31:1534-44.

19. Ibanez L, Ong K, Dunger DB, de Zegher F. Early development of adiposity and insulin resistance after catch-up weight gain in small-for-gestational-age children. J Clin Endocrinol Metab 2006;91:2153-58.

20. Corvalán C, Uauy R, Stein AD, Kain J, Martorell R. Effect of growth on cardiometabolic status at 4 y of age. Am J Clin Nutr 2009;90:547-55.

21. Stein CE, Fall CH, Kumaran K, Osmond C, Cox V, Barker DJ. Fetal growth and coronary heart disease in south India. Lancet 1996;348:1269-73.

22. Barker DJP, Osmond C, Forsén TJ, Kajantie E, Eriksson JG. Trajectories of growth among children who have coronary events as adults. N Engl J Med 2005;353:1802-9.

23. Wells JCK, Fewtrell MS. Is body composition important for paediatricians? Arch Dis Child 2008;93:168-72.

24. Wells JCK, Chomtho S, Fewtrell MS. Programming of body composition by early growth and nutrition. The Proceedings of the Nutrition Society 2007;66:423-34.

25. Yajnik CS, Fall C, Coyaji KJ, Hirve SS. Neonatal anthropometry: the thin–fat Indian baby. The Pune maternal nutrition study. Int J Obes 2003:173-80.

26. Popkin BM. The nutrition transition and obesity in the developing world. The Journal of Nutrition 2001;131:871S-3S.

27. Andersen GS, Girma T, Wells JCK, Kæstel P, Michaelsen KF, Friis H. Fat and fat-free mass at birth: air displacement plethysmography measurements on 350 Ethiopian newborns. Pediatr Res 2011;70:501-6.

28. Andersen GS, Girma T, Wells JC, Kæstel P, Leventi M, Hother AL, Michaelsen KF, Friis H. Body composition from birth to 6 mo of age in Ethiopian infants: reference data obtained by air-displacement plethysmography. Am J Clin Nutr 2013;98:885-94.

29. Grijalva-Eternod CS, Wells JC, Girma T, Kaestel P, Admassu B, Friis H, Andersen GS. Midupper arm circumference and weight-for-length z scores have different associations with body composition: evidence from a cohort of Ethiopian infants. Am J Clin Nutr 2015;102:593-9.

30. Wells JCK, Fuller NJ, Dewit O, Fewtrell MS, Elia M, Cole TJ. Four-component model of body composition in children: density and hydration of fat-free mass and comparison with simpler models. Am J Clin Nutr 1999;69:904-12.

31. Wells JCK, Fuller NJ, Wright A, Fewtrell MS, Cole TJ. Evaluation of air-displacement plethysmography in children aged 5-7 years using a three-component model of body composition. The British journal of nutrition 2003;90:699-707.

32. Fields DA, Goran MI, McCrory MA. Body-composition assessment via air-displacement plethysmography in adults and children: a review. Am J Clin Nutr 2002;75:453-67.

33. Seigler L, Wu WT. Separation of serum high-density lipoprotein for cholesterol determination: ultracentrifugation vs precipitation with sodium phosphotungstate and magnesium chloride. Clin Chem 1981;27:838-41.

34. Friedewald WT, Levy RI, Fredrickson DS. Estimation of the concentration of low-density lipoprotein cholesterol in plasma, without use of the preparative ultracentrifuge. Clin Chem 1972;18:499-502.
