# Supplementary material for: Associations of fat mass and fat-free mass accretion in infancy with body composition and cardiometabolic risk markers at 5 years: The Ethiopian iABC birth cohort study
Source: PLoS Med. 2019 Aug 20;16(8):e1002888. doi: 10.1371/journal.pmed.1002888 (PMC6701744; doi:10.1371/journal.pmed.1002888)
Supplement: S3 Text — (PDF) [file pmed.1002888.s012.pdf]

S3 Text

Detailed description of the linear-spline mixed-effects modelling

We estimated child-specific fat mass (FM) and fat-free (FFM) at birth and child-specific growth velocities in selected age periods using a linear-spline mixed effects (LSME) modelling approach, as described elsewhere [1-5]. The estimated model parameters include both fixed and random effects. The fixed effect parameters of the LSME models were the predicted study population average at birth (model intercept) and growth velocities in the selected age periods (slope coefficients). The random effect parameters were the child-specific random intercepts and slopes, expressing the individual deviations from the predicted study population average at birth and growth velocities in the selected age intervals, respectively. The length of the specific age intervals over which FM and FFM growth velocities are quantified is determined by a selected number of knot points that separates each growth velocity curve. The LSME model specification allows for the slopes of each fixed and random growth curve to vary in each growth interval, with the restriction that each growth curve must join together at the age specified by the knot point(s).

For example, a LSME model that estimates linear changes in FM or FFM in a number of age periods separated by c knot points was specified as follows:

We define c knot points at times t_k, k = 1,…, c. For child i with y_{ij} (e.g. FM or FFM) observed at age t_{ij} we create c splines denoted s_{ijk}:

$$s_{ijk} = 0 \qquad ,if\ t_{ij} \leq t_k$$

$$s_{ijk} = t_{ij} - t_k \quad ,if\ t_{ij} > t_k$$

For instance, if the model is specified with a knot point at t_k = 3 months, the spline s_{ijk} is zero for ages below or equal to 3 months, but above zero for ages above 3 months, and defined

as number of months after 3 months. Hence, at age one months the spline s_{ijk} would be zero, but at four months it would be one months.

Thus, the model was specified as follows:

$$y_{ij} = \beta_0 + u_{i0} + \sum_{k=1}^{c} (\beta_k + u_{ik}) s_{ijk} + e_{ij} ,$$

where y_{ij} is the FM or FFM of child i at age j. $\beta_0, \beta_1, \ldots, \beta_c$ are the fixed effects parameters which describes the average intercept and slopes between each set of knot points. Hence, the slope between knots *k-1* and *k* is the sum of $\beta_1, \beta_2, \ldots, \beta_{k-1}$. The term, u_{i0} is the random intercept which describes the deviation of child i's intercept from the average intercept, and u_{ik} are the random slopes which describes the deviations for child i from the average slopes. e_{ij} are the occasion level residuals (i.e. the difference between observed and estimated values for child i at age j).

To approximate the non-linear relationship of FM and FFM as a function of age, we specified a LSME model with linear base splines with one knot point. To identify the best placement of the knot point, we ran a series of models with knot points placed at different ages with a high data density (i.e. 1.5, 2, 2.5, 3, 3.5, 4, and 4.5 months), as suggested by Howe et al. [1]. For the modelling of FM growth, a model with a knot point at 3 months of age yielded the lowest Bayesian information criterion (BIC) value and was therefore selected as the best fitting model. For the modelling of FFM growth, a model with a knot point at 2 months of age yielded the lowest Bayesian information criterion (BIC) value, followed by a model with a knot point at 2.5 and 3 months of age. However, in order to assess the differential effect of infant FM and FFM growth on body composition and cardiometabolic risk marker at 5 years

of age from similar periods, we selected a model with a knot point at 3 months of age to estimate FFM growth.

The LSME model was specified using the 'lmer' function in the R-package 'lm4' (version 1.1.17) in R (version 3.4.1) [6]. An example of the R specification of the FM model above is:

```
lmer( formula = Fat mass (kg) ~ Age in months + pmax((Age in months) − 3, 0) +
    (Age in months + pmax((Age in months) − 3, 0) | Id) ,
    REML = FALSE,
    na.action = na.exclude,
    data = Data)
```

where the fixed term in the model specification included the response variable '*Fat mass (kg)*' and the covariate 'Age in months' specified with linear base splines using the R function 'pmax()' from the R Base Package. The random term was specified similar to the fixed term of the model. Thus, as shown in **Fig 2.,** the slopes of the child-specific growth curves were allowed to deviate from the average growth curve of the study population. **S4 Fig.** shows matrices of the model assumptions tests for the estimation of FM and FFM growth velocity.

References

1. Howe LD, Tilling K, Matijasevich A, Petherick ES, Santos AC, Fairley L, et al. Linear spline multilevel models for summarising childhood growth trajectories: A guide to their application using examples from five birth cohorts. Stat Methods Med Res. 2016; 25: 1854–1874. doi: 10.1177/0962280213503925.

2. Tilling K, Macdonald-Wallis C, Lawlor DA, Hughes RA, Howe LD. Modelling childhood growth using fractional polynomials and linear splines. Ann Nutr Metab. 2014; 65: 129-138. doi: 10.1159/000362695.

3. Naumova EN, Must A Fau - Laird NM, Laird NM. Tutorial in Biostatistics: Evaluating the impact of 'critical periods' in longitudinal studies of growth using piecewise mixed effects models. Int J Epidemiol. 2001; 30: 1332–1341.

4. Hardy R. Commentary: Are piecewise mixed effects models useful in epidemiology? Int J Epidemiol. 2001; 30: 1341-1342. doi: 10.1093/ije/30.6.1341.

5. Tu YK, Tilling K, Sterne JA, Gilthorpe MS. A critical evaluation of statistical approaches to examining the role of growth trajectories in the developmental origins of health and disease. Int J Epidemiol. 2013; 42: 1327-1339. Epub 2013/09/17. doi: 10.1093/ije/dyt157.

6. Bates D, Mächler M, Bolker B, Walker S. Fitting Linear Mixed-Effects Models Using lme4. J Stat Softw. 2015; 67: 1-48. doi: 10.18637/jss.v067.i01.
